# Brain mechanisms underlying neuropsychiatric symptoms in Alzheimer’s disease: a systematic review of symptom-general and –specific lesion patterns

**DOI:** 10.1186/s13024-021-00456-1

**Published:** 2021-06-07

**Authors:** Yaojing Chen, Mingxi Dang, Zhanjun Zhang

**Affiliations:** 1 State Key Laboratory of Cognitive Neuroscience and Learning, Beijing Normal University, Beijing, 100875 China; 2 BABRI Centre, Beijing Normal University, Beijing, 100875 China

**Keywords:** Neuropsychiatric symptoms, Alzheimer’s disease, Neuroimaging, Brain lesion pattern, Brain circuit

## Abstract

Neuropsychiatric symptoms (NPSs) are common in patients with Alzheimer’s disease (AD) and are associated with accelerated cognitive impairment and earlier deaths. This review aims to explore the neural pathogenesis of NPSs in AD and its association with the progression of AD. We first provide a literature overview on the onset times of NPSs. Different NPSs occur in different disease stages of AD, but most symptoms appear in the preclinical AD or mild cognitive impairment stage and develop progressively. Next, we describe symptom-general and -specific patterns of brain lesions. Generally, the anterior cingulate cortex is a commonly damaged region across all symptoms, and the prefrontal cortex, especially the orbitofrontal cortex, is also a critical region associated with most NPSs. In contrast, the anterior cingulate-subcortical circuit is specifically related to apathy in AD, the frontal-limbic circuit is related to depression, and the amygdala circuit is related to anxiety. Finally, we elucidate the associations between the NPSs and AD by combining the onset time with the neural basis of NPSs.

**Supplementary Information:**

The online version contains supplementary material available at 10.1186/s13024-021-00456-1.

## Background

As the worldwide population ages, over 50 million people are now living with dementia, and this number is set to increase to 152 million by 2050 [1]. Dementia has reached epidemic proportions, with major social, medical, and economic burdens [2]. The leading cause of dementia is Alzheimer’s disease (AD), whose main clinical manifestation is cognitive impairment, but 80% of AD patients also show various behavioural and psychological symptoms, collectively known as neuropsychiatric symptoms (NPSs) [3]. These symptoms are associated with more rapid progression to severe dementia and an earlier death [4]. They also adversely reduce the quality of life of patients and caregivers [5].

Cross-sectional and longitudinal studies have indicated that different NPSs occur mostly at different stages of AD [6–8]. For example, hallucinations seem to be more common in patients with severe AD, while irritability tends to occur in the early stages of the disease [7]. Even if some NPSs seem to appear together and share some of the same pathological features (for example, both depression and apathy are expressed as loss of interest and motivation), they have different pathological mechanisms. A clear understanding of the pathological mechanisms of differential NPSs is crucial for the early detection and treatment of the disease and the NPSs.

Many empirical studies have been conducted to understand the neural pathogenesis of NPSs in AD and its role in the progression of AD primarily using neuroimaging techniques. Yet, there has only been a small, though growing, number of reviews on this body of literature. Moreover, most of these reviews were merely of qualitative with regard to the brain regions associated with NPSs [9–12]. In our current efforts, we attempted to provide a more comprehensive review focusing on the quantitative aspects of relevant reports available in the literature. In doing so, we will manage to summarize the number of significant associations between NPSs and brain regions to describe quantitatively symptom-general and -specific patterns of brain lesions, so that we can determine the core damage regions of each symptom, with more detailed pathological information of NPSs in AD.

Accurate assessments of NPSs are the basis of NPSs pathogenesis neuroimaging research. Although these assessments are well-established and available in the NPSs literature with or without the use of neuroimaging, reviews of NPSs have only briefly summarized these NPSs test [13, 14]. We believe that a more detailed description of these tests is needed, including the applicable population of the tests, their advantages and disadvantages, among others. More importantly to the study of AD, we cannot ignore the problem of assessing NPSs in AD patients, accurate assessment of whose neurological and behavioural symptoms is critical and yet compounded with NPSs. Finally, a number of researches have shown that the onset time and the association of NPSs with different cognitive domains are variable, better understanding of which helps to comprehend the association between NPSs and AD. To the best of our knowledge, such issues have not been systematically summarized in review.

Overall, the current understanding of the pathological mechanism of NPSs in patients with AD is limited, especially the relationship between NPSs and AD, so a systematic review is needed to clarify these problems. We therefore provide an extensive review to 1) summarize the clinical assessment, onset time, and association with cognitive impairment of NPSs, and 2) quantitatively describe symptom-general and -specific patterns of brain lesions and brain circuits; and 3) elucidate the associations between the NPSs and AD.

## Main text

### Methods

#### Search strategy and selection criteria

We performed a systematic literature review following the guidelines for Preferred Reporting Items for Systematic Reviews and Meta-Analyses (PRISMA, http://www.prisma-statement.org/). Research papers published up to March 2020 were identified in the databases PubMed and PsycINFO databases, using the following terms: ‘Alzheimer disease’ or ‘mild cognitive impairment’ AND ‘neuropsychiatric symptoms’, ‘apathy’, ‘delusions’, ‘depression’, ‘agitation’, ‘hallucination’, ‘anxiety’, ‘euphoria’, ‘disinhibition’, ‘irritability’, ‘aberrant motor behavior’, ‘sleep disturbances’, ‘appetite disturbances’, or ‘eating disorder’. The selection criteria were as follows: (1) To limit the heterogeneity, we focused only on AD and excluded studies that included non-AD dementia and non-amnesic MCI; (2) To ensure the reliability of the research results, articles with a sample size less than 10 were excluded; (3) Articles with subjects younger than 50 years of age were excluded.

#### Study quality assessment

All included original manuscripts were assessed by two independent reviewers to avoid possible bias and reporting quality using the Joanna Briggs Institute-Qualitative Assessment and Review instrument (JBI-QARI) [15]. Six papers were considered low quality and were excluded. Twenty-five papers comparing the pathological mechanisms of subjects with and without NPSs, without matching confounding variables such as age and cognitive ability, but without methodological problems, were rated as of moderate quality. The rest of the papers were rated as high quality with low bias.

Finally, 114 studies were included, including 66 neuroimaging studies to explore the pathological mechanism of NPSs and 48 non-imaging studies to summarize clinical assessments of NPSs (*N* = 25), onset times of NPSs (*N* = 8), and the associations of NPSs with cognitive impairment (*N* = 15). sFigure 1 summarizes the process for study selection and inclusion. AD is defined by the standard diagnostic criteria, primarily NINCDS-ADRDA (approximately 78%), followed by the CDR, DSM-IV, and CERAD. Amnestic MCI (aMCI) is defined by Petersen criteria.

### Neuropsychiatric symptoms in Alzheimer’s disease

In this section, we will first summarize the existing clinical assessment scales of NPSs and discuss the main problems these assessments have when used to measure NPSs in AD patients. Then, we will summarize the results on the onset times of NPSs. Final, we will summarize the report findings on the association between NPSs and the cognitive impairment.

#### Clinical assessment of neuropsychiatric symptoms

We summarized the scales for measuring neuropsychiatric and behavioural symptoms, listed the measured symptoms, applicable population, and described each scale in Table 1. Multiple instruments are available for assessing NPSs in AD, but there are several problems that need to attention when selecting a measurement scale. First, since NPSs overlap with dementia symptoms, attempts should be made to rule out the effects of cognitive impairment on the measures. For example, the Behavioral Pathology in Alzheimer’s Disease Rating scale (BEHAVE-AD) is commonly used to assess non-cognitive behavioural disorders in patients with AD [16]. Secondly, some scales are only applicable to subjects with a certain level of cognition. For example, the Hamilton depression scale (HAMD) is only used to assess patients with mild dementia [17]; while the Depressive Signs Scale (DSS) cannot assess depressive symptoms in patients with mild or moderate dementia [18]. In addition, multiple NPSs tend to occur simultaneously (e.g. apathy and depression), and the functional relationships among these different NPSs are not clear. It is recommended to adopt a scale that can measure multiple NPSs simultaneously, and all of them should be independent measurements, such as Neuropsychiatric Inventory (NPI) scale [19]. Finally, the patient and the caregiver may exaggerate or conceal the severity of the symptoms because of the pathological injury and the caregiver’s emotions, respectively, so the instrument should be graded based on information from as many sources as possible, such as the Dimensional Apathy Scale [20].

**Table 1:** Summary of clinical assessment scales for neuropsychiatric symptoms

Measured symptoms	Scale	Applicable population	Scale description
Delusions, hallucinations, anxiety, agitation, euphoria, disinhibition, irritability, apathy, aberrant motor behavior, sleep and eating disturbance	Neuropsychiatric Inventory	Generally applicable	Assessment of broader psychopathology; Collect information that may distinguish the different causes of dementia.
Paranoid and delusional ideation, hallucinations, activity disturbances, aggressiveness, diurnal rhythm disturbances, affective disturbances, anxieties and phobias	Behavioral Pathology in Alzheimer’s disease rating scale	AD	Specifically for patients with AD, excluding the effects of cognitive impairment on measurement.
Anxiety, depression, aberrant motor behavior, delusions and hallucinations, disturbance of consciousness	Behavior Rating Scale for Dementia	AD	Detailed content; High variability and sensitivity
Psychotic disorders, mood disorders, substance use disorders, anxiety disorders, etc	Diagnostic and Statistical Manual of Mental Disorders	Generally applicable	The scale included multidimensional and single-dimensional assessments. There were three self-assessment versions: the adult, the child/adolescent, and the parent/guardian.
Mental health, walking, eating, diurnal rhythm, aggressive behavior, sexual behavior, incontinence, individual behavioral abnormalities	Present Behavioral Examination	Generally applicable	Interviews were conducted with primary caregivers for patients with dementia or other neuropsychiatric disorders. It assesses behavior over the preceding 4 weeks.
Motor, intellectual and emotional functions and different symptoms characteristic for dementia.	Gottfries–Brane–Steen scale	Generally applicable	It can measure changes in dementia symptoms over a certain amount of time and evaluate the effect of treatment.
Apathy (Unidimension)	Apathy Evaluation Scale	AD/PD/ stroke	Three versions of the AES (clinician, informant, and self-rated) were used to evaluate the emotional apathy of patients in the past 4 weeks.
	Dementia Apathy Interview and Rating	AD	Attempt to differentiate limited activity and engagement due to lack of interest from the inability or longstanding, premorbid personality traits through question construction, and interview format.
	Apathy Inventory	AD/PD/MCI	It consists of two sets of questionnaires, one for caregivers and the other for patient-based assessments. Each problem involves frequency and severity.
Apathy (Multidimension)	Dimensional Apathy Scale	Generally applicable	A comprehensive and robust measure of multidimensional apathy
	Lille Apathy Rating Scale	PD	The scale is based on a structured interview, including 33 items, divided into nine domains. Responses are scored on a dichotomous scale.
	Apathy Motivation Index	Healthy people	Identified subtypes of apathy in behavioral, social, and emotional domains.
Depression	Hamilton Depression Scale	Mild AD	Emphasis on patient perception and memory; only appropriate for evaluating patients with mild dementia
	Cornell Scale for Depression in Dementia	AD	Accurately distinguish depressive symptoms in AD patients from their cognitive dysfunction
	Depressive Signs Scale	Severe dementia	Can not assess depressive symptoms in patients mild or moderate dementia.
	The Center for Epidemiologic Studies Depression Scale	Generally applicable	The scale is a short self-report scale designed to measure depressive symptomatology in the general population. More emphasis is placed on the individual’s emotional physical examination, less on the somatic symptoms of depression.
	Montgomery-Asberg Depression Rating Scale	People with depression	A clinical interview with ten items, each scored on a scale from 0 to 6, particularly sensitive to treatment effects.
	Geriatric depression scale	Elderly with depression	More sensitively examine somatic symptoms specific to older depressed patients, with 30 core items.
Anxiety	Hamilton Anxiety Scale	Generally applicable	Can not distinguish depression and anxiety well; The assessment of AD depression lacks specificity
	Worry Scale	Mild dementia/ non-dementia adults	The Worry Scale is a brief, unidimensional scale with good reliability and concurrent validity.
	Rating Anxiety in Dementia	Dementia	The items in the scale were rated according to the person’s symptoms and signs of anxiety over the previous 2 weeks, including worry, sleep disturbance, irritability, and a number of somatic symptoms
Depression, Anxiety	Hospital Anxiety and Depression Scale	Adults	It contains two subscales of anxiety and depression, each with seven questions. The psychiatric assessment for each patient lasted about 20 min.
Aggressive	Rating Scale For Aggressive Behavior in the Elderly	Elderly	Not only to assess patients in nursing homes or hospitals, but also in the community
	Cohen-Mansfield Agitation Inventory	Generally applicable	The CMAI is a caregivers’ rating questionnaire consisting of 29 agitated behaviors, each rated on a 7-point scale of frequency. A disruptiveness scale was added to later versions.
Sleep disturbance	Women’s Health Initiative Insomnia Rating Scale	Women	A brief, five-item scale evaluating the frequency and intensity of certain sleep difficulties in respondents and requiring between 2 and 5 min

*Abbreviations*: *AD* Alzheimer’s disease, *MCI* mild cognitive impairment, *MMSE* Mini-mental State Examination, *PD* Parkinson’s disease

Therefore, although many scales have been developed to measure neuropsychiatric and behavioural symptoms, scales that can accurately measure different NPSs in patients with AD are still inadequate or lacking. In practice, we should carefully select appropriate scales according to the population to be assessed and their needs.

#### The onset time of neuropsychiatric symptoms

**Fig. 1:**
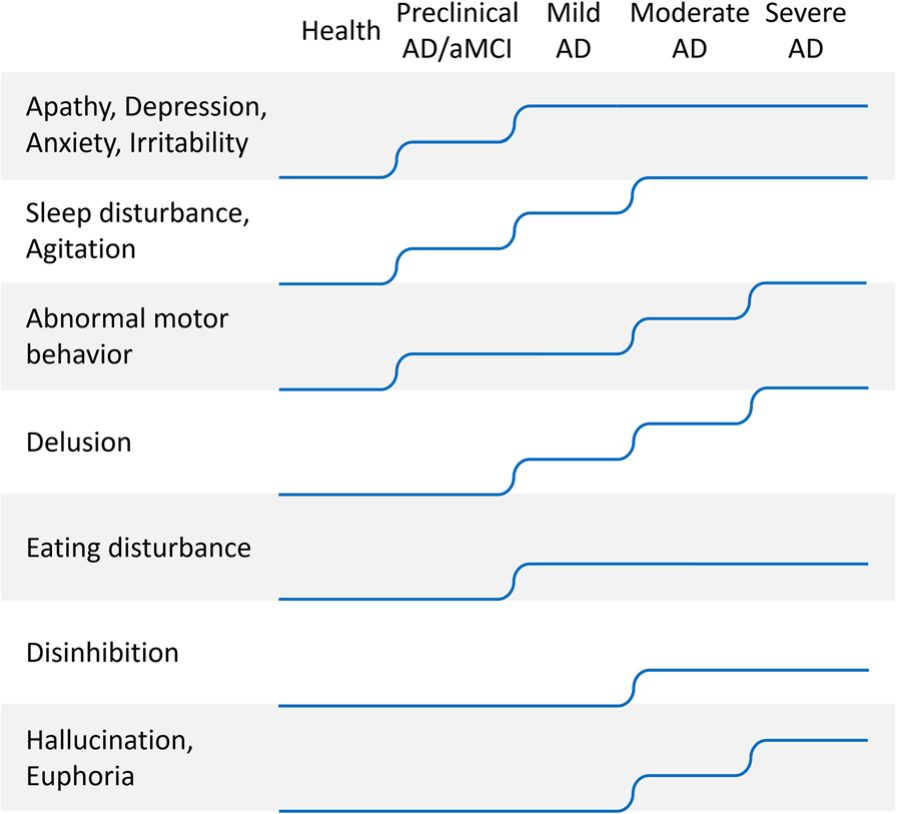
Schematic diagram of the onset time of NPSs. Disease progression is divided into five stages: healthy stage, preclinical AD or aMCI, mild AD, moderate AD, and severe AD. Each rise of the curve represents a significant increase in the prevalence of the NPSs compared to the previous stage. Abbreviations: AD, Alzheimer’s disease; aMCI, amnestic mild cognitive impairment

Table 2 summarizes the study findings that provide information on the onset time of NPSs. For example, one study suggested that the prevalence of delusion was significantly increased in mild AD compared to aMCI [21]; and another study showed that the prevalence of aberrant motor behaviours, delusion, hallucinations and sleep disturbances were significantly higher in moderate AD than in mild AD [22]. For the purpose of this study, the AD continuum was divided into four stages as aMCI (or preclinical AD), mild AD, moderate AD, and severe AD, and these findings are summarized in sTable 1 and Fig. 1. sTable 1 shows that several studies have found that the prevalence of NPSs are significantly higher at a certain stage than at its previous one. Each rise of the curve in Fig. 1 represents at least one study suggesting a significant increase in the prevalence of the NPSs compared to the previous stage.

**Table 2:** The onset time of neuropsychiatric symptoms: study characteristics

Source	Stages	Contrast	Findings
Craig, 2005 [7]	Probable AD	MMSE: < 10 vs 10–20 vs > 20	Depression and apathy were the earliest to appear, and hallucinations, euphoria, and aberrant motor behavior were the latest symptoms to emerge. Hallucinations were significantly more common in severe dementia (MMSE< 10). Irritability was most prevalent in early disease (MMSE> 20).
Cheng, 2012 [22]	AD	Moderate AD vs mild AD	The prevalence of aberrant motor behavior, delusion, hallucination and sleep disturbance was significantly higher in moderate AD than in mild AD.
Burns, 1990 [23]	AD	Severe AD vs moderate AD	The prevalence of aberrant motor behavior and sexual disinhibition was significantly higher in severe AD than in moderate AD.
Hwang, 2004 [21]	aMCI/ Mild AD	aMCI vs controls; mild AD vs aMCI	There were significant differences in apathy, irritability, anxiety, agitation and abnormal motor behavior between the aMCI and controls. Delusion was significantly increased in mild AD compared to aMCI.
Iulio, 2010 [24]	aMCI/AD	aMCI vs controls;	The prevalence of depression, apathy, agitation and irritability was significantly higher in aMCI than in normal controls.
Ehrenberg, 2018 [25]	AD	Braak I/II, Braak III/IV, Braak V/VI vs controls	In Braak I/II, significantly increased odds were detected for agitation, anxiety, appetite changes, depression, and sleep disturbances, compared to controls. Increased odds of agitation continue into Braak III/IV. Braak V/VI is associated with higher odds for delusions.
Jost, 1996 [6]	AD	Time order	Apathy, depression, sleep disturbance and anxiety appeared before the diagnosis of AD. Irritability and delusions occurred within 5 months after diagnosis; Inappropriate sexual behavior, wandering, agitation within 5–10 months after diagnosis; Hallucination and aggression appear 10 months after diagnosis.
Linde, 2016 [26]	AD	Persistence	Apathy and abnormal behavior showed high persistence; Irritability, agitation, depression and anxiety showed moderate persistence; Delusions, hallucination, appetite changes, and sleep disturbance showed short persistence.

*Abbreviations*: *AD* Alzheimer’s disease, *aMCI* amnestic mild cognitive impairment, *NPSs* neuropsychiatric symptoms, *MMSE* Mini-mental State Examination

Most NPSs occur in the preclinical AD or aMCI phase, including apathy, depression, anxiety, irritability, agitation, sleep disturbances, and abnormal motor behaviour (Fig. 1, sTable 1). Delusions and eating disturbances occur in the mild AD phase. Finally, disinhibition, hallucinations, and euphoria occur in the moderate AD phase. In addition to disinhibition and appetite changes, the onset of the other NPSs is progressive, meaning the prevalence of these symptoms continues increasing as the disease progresses.

#### Neuropsychiatric symptoms and cognitive dysfunction in AD

By collecting and sorting out the literature findings on the association between NPSs and cognitive decline, we found that NPSs were closely related to global cognitive impairment [27, 28] and activities of daily living decline [29, 30]. In general, different NPSs in patients with AD were related to specific cognitive impairment (Table 3, sTable 2). Mental symptoms and agitation seem to be associated with more cognitive domains and more rapid cognitive decline, all accompanied by impaired language and memory function [28, 29, 31–34]. In addition, delusions are associated with decreased executive function, reasoning ability, and conceptualization [31]; hallucinations are associated with decreased visuospatial function [33]; while agitation is associated with decreased executive function, visuospatial function, and conceptualization [29, 32, 35]. Apathy is closely related to executive function [29, 36–38] and comportment (which stands for Social Behavior) [29, 32]. Depression are accompanied by a decline in executive [38, 39] and memory function [39, 40]. Abnormal motor behaviour is associated with executive and language impairment. Disinhibition and sleep disturbances are related only to executive and memory functions, respectively [29, 35, 41]. However, we did not find that euphoria, irritability, or eating disturbances were associated with any specific cognitive domains. Hence, executive function is the most closely related to NPSs among all cognitive domains, implying the executive function deficits and some NPSs may stem from common neurobiological mechanisms.

**Table 3:** The relationship between NPSs and cognitive dysfunction: study characteristics

Source	Participants (Number)	Findings
Senanarong, 2005 [29]	AD (*N* = 73)	Clock-drawing test correlated with agitation, apathy, and disinhibition; Verbal Fluency correlated with agitation; Activities of Daily Living and Functional Assessment Questionnaire scores correlated with agitation, apathy, and disinhibition; Comportment predicted total NPI-12 score and apathy; Memory predicted agitation/aggression.
McPHERSON, 2002 [36]	AD (*N* = 80)	AD patients with apathy performed significantly worse on tests of executive function (WAIS–R Digit Symbol, Trail-Making, Stroop Color Interference Test) than AD patients without apathy.
Grossi, 2013 [37]	AD (*N* = 32)	The apathetic AD had poorer performance than non-apathetic AD on frontal tasks (Inverse Motor Learning test).
Jeste, 1992 [31]	AD (*N* = 107)	Patients with delusions were significantly more impaired than those without delusions on the MMSE, Blessed Information-Memory-Concentration Test, Dementia Rating Scale (especially its conceptualization and memory subtests), verbal fluency, modified Wisconsin Card Sorting Test, and the Similarities subtest of the Wechsler Adult Intelligence Scale-revised.
Son, 2013 [40]	AD (*N* = 49)	Seventeen AD patients with depression versus 32 patients with dementia only showed decreased immediate recall for a word list and constructional praxis scores.
Scarmeas, 2005 [42]	Early AD (*N* = 456)	Delusions and hallucinations predict cognitive (Columbia MMSE score) and functional (Blessed Dementia Rating Scale score) decline.
Boyle, 2003 [43]	AD (*N* = 45)	Apathy correlated with Activities of Daily Living.
Chen, 1998 [35]	AD (*N* = 31)	Deficits in four executive skills tests were significantly associated with the Agitation/Disinhibition factor score and total neuropsychiatric score on the Neurobehavioral Rating Scale, as well as the Activities subscore on the Blessed Dementia Scale.
Sultzer, 2014 [34]	AD (*N* = 88)	Patients with delusions had lower Dementia Rating Scale memory subscale scores.
Westerberg, 2010 [41]	aMCI (*N* = 10)	Inadequate memory consolidation in aMCI patients is related to declines in subjective sleep indices.
Rozum, 2017 [32]	Severe dementia (*N* = 89)	Comportment (Social Behavior) was correlated with Apathy, while conceptualization (Sorting by Color), language (Naming, Comprehension), memory (Remote Recall, Learning), and visuospatial ability (Figure Tracing, Drawing) were each correlated with agitation/aggression. Comportment and memory were associated with total NPI-12.
Nagata, 2010 [44]	AD (*N* = 75)	Aberrant motor behaviors correlated with Frontal Assessment Battery total and the subtest scores (lexical fluency, conflicting instructions).
Wilson, 2000 [33]	AD (*N* = 410)	Compared with AD patients without hallucination, the average annual rate of decline was increased about memory, visuoconstruction, repetition, and naming in those with hallucination.
Lopez, 1991 [28]	AD (*N* = 17)	AD patients with delusions and hallucinations had a more rapid rate of decline, as measured by the MMSE, a specific defect in receptive language, and a greater frequency of aggression and hostility.
Nakaaki, 2008 [38]	AD (*N* = 88)	Total Frontal Assessment Battery scores differed significantly between the AD patients with depression/apathy and those without depression/apathy.
Onofrio, 2012 [30]	AD (*N* = 166)	A significant association was also found between the impairment of the instrumental activities of daily living and agitation/aggression, anxiety, aberrant motor activity, depression, apathy, irritability/lability, sleep and eating disturbances in AD.

*Abbreviations*: *AD* Alzheimer’s disease, *aMCI* Amnestic mild cognitive impairment, *MMSE* Mini-mental State Examination, *N* number, *NPI* Neuropsychiatric Inventory, *Aβ* Amyloid-β

### Neuroimaging findings in neuropsychiatric symptoms

**Fig. 2:**
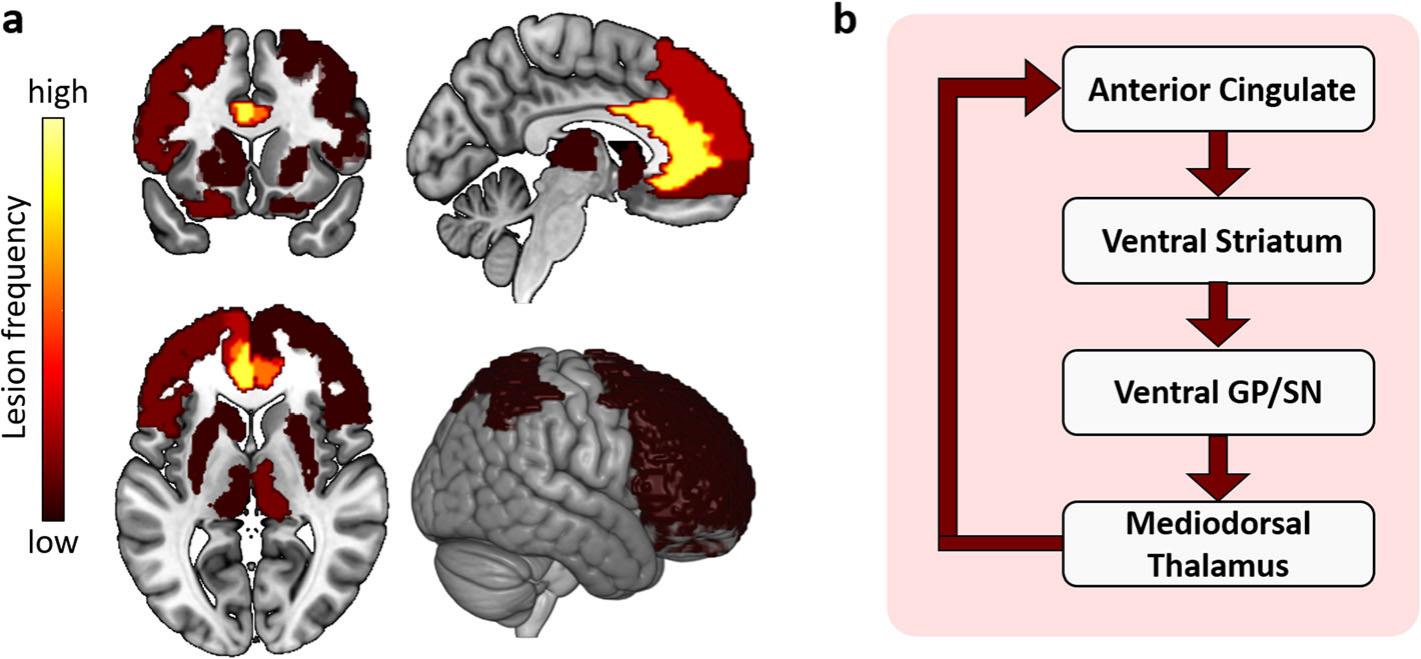
The brain lesion pattern and anterior cingulate-subcortical circuit of apathy. **a** The lesion brain region with the highest frequency of apathy is the anterior cingulate cortex (especially on the left), followed by the left medial frontal, medial orbitofrontal, medial thalamus, left lateral orbitofrontal, left superior and ventrolateral frontal regions, as well as the parietal, the head of the left caudate nucleus, putamen, and other regions of the frontal lobe. **b** The anterior cingulate-subcortical circuit begins in the anterior cingulate cortex and projects to the ventral striatum, which includes the nucleus accumbens, ventral putamen, ventromedial caudate, and olfactory tubercle. The ventral striatum has circuit linkages to the ventral pallidum and rostrodorsal substantia nigra. Then the ventral pallidum provides limited input to the mediodorsal thalamus. The anterior cingulate circuit is closed with projections from the dorsal portion of the magnocellular mediodorsal thalamus to the anterior cingulate. Abbreviations: GP: globus pallidus, SN: substantia nigra. **b** A visual adaptation of a figure from Nobis et al. [13], with permission

**Fig. 3:**
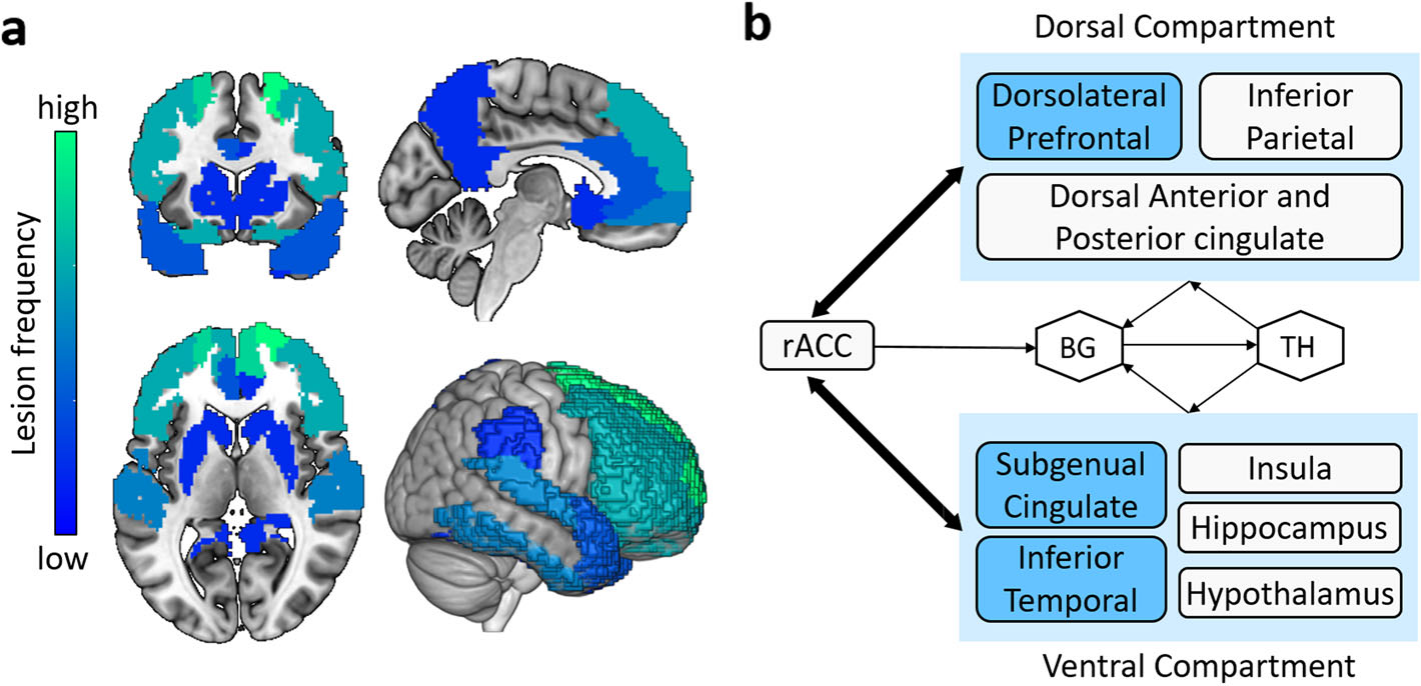
The brain lesion patterns and frontal-limbic circuit of depression in AD. **a** The brain region with the highest frequency of depression lesions is the superior frontal lobe, followed by the left inferior temporal and other frontal regions, as well as other temporal regions, the anterior cingulate, entorhinal, right hippocampal, caudate nucleus, lentiform nucleus, fusiform, right posterior cingulate, precuneus, supramarginal and parietal lobe. **b** The frontal-limbic circuit is composed of a dorsal part dominated by the dorsolateral prefrontal cortex and ventral part dominated by the subgenual cingulate and inferior temporal cortex. A direct projection from the subgeniual cingulate to the dorsolateral prefrontal cortex and a bidirectional indirect pathway through multiple marginal regions, including the posterior cingulate, hypothalamus, hippocampus, and insula are delineated. Abbreviations: rACC = rostral anterior cingulate; BG = basal ganglia; Th = thalamus. **b** A visual adaptation of a figure from Mayberg et al. [99], with permission

**Fig. 4:**
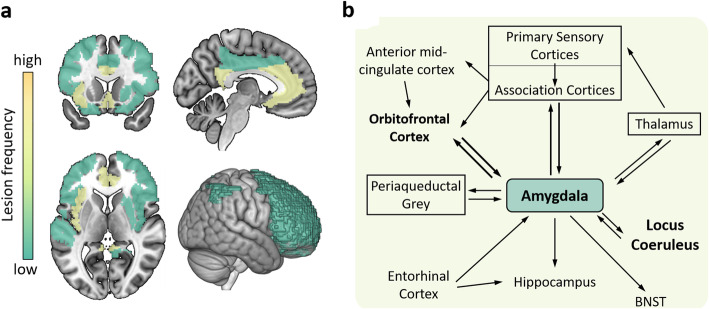
The brain lesion pattern and amygdala circuit of anxiety in AD. **a** The anterior and posterior cingulate cortex, entorhinal cortex, parahippocampal gyrus and insula cortex are the highest frequency of anxiety lesion regions, and the second is the amygdala, right precuneus, inferior parietal lobule, left anterior superior temporal, putamen, middle cingulate cortex and the frontal lobe. **b** The afferent arm of the anxiety circuit includes the exteroceptive sensory systems of the brain, which convey the sensory information contained in anxiety-inducing stimuli to the dorsal thalamus. An exception is the olfactory system, which carries information through the amygdala and entorhinal cortex, not the thalamus. Visceral afferent pathways alter the function of the locus coeruleus and amygdala. The thalamus relays sensory information to the primary sensory receptive areas of the cortex, which project to adjacent unimodal and polymodal cortical association areas. The cortical association areas send projections to the amygdala, entorhinal cortex, orbitofrontal cortex, and cingulate gyrus. The efferent pathways involving the amygdala, locus coeruleus, hypothalamus, periaqueductal gray, and striatum mediate autonomic, neuroendocrine, and skeletal-motor responses are associated with anxiety. Abbreviations: BNST = bed nucleus of the stria terminalis. **b** A visual adaptation of a figure from Charney et al. [100], with permission

**Fig. 5:**
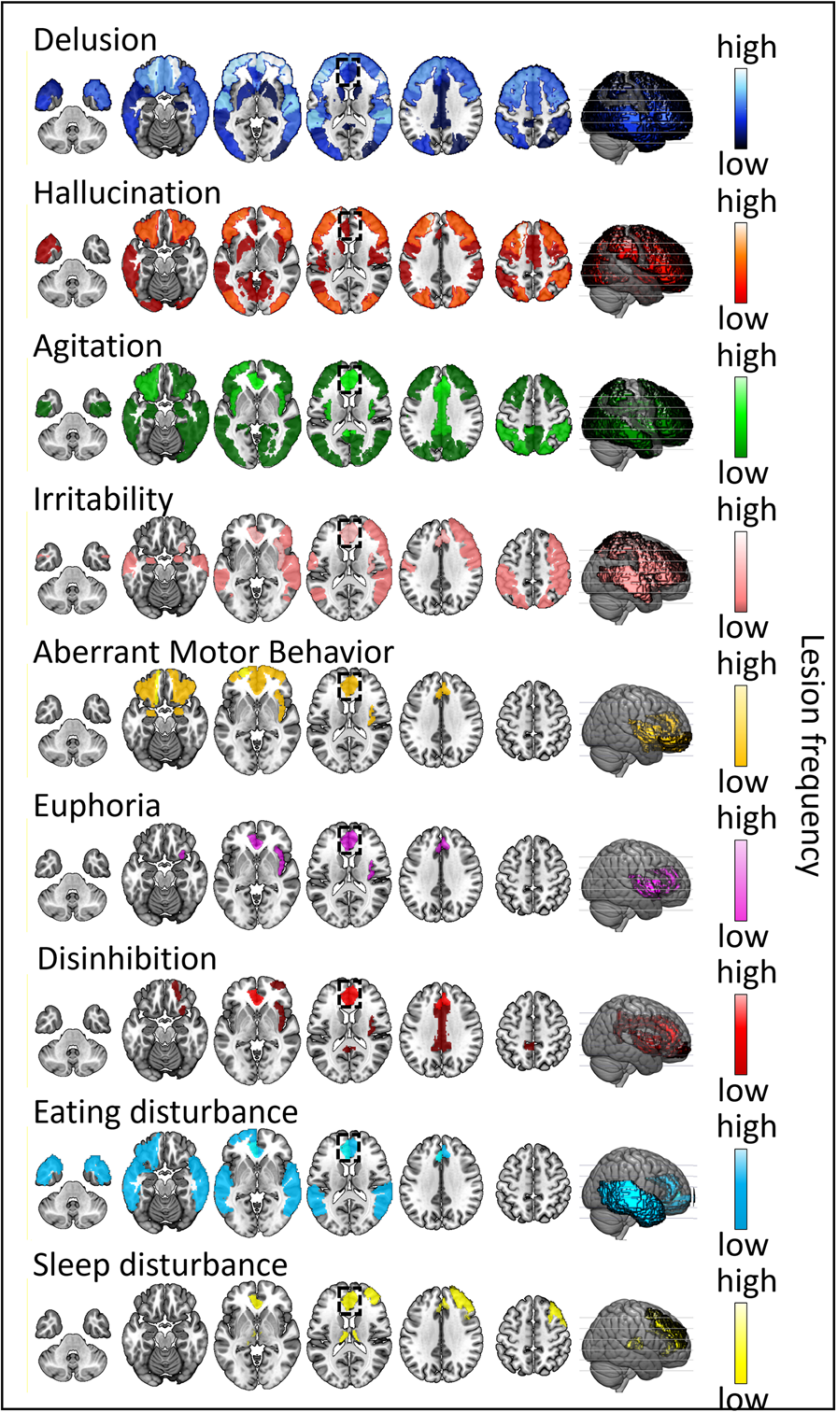
The brain lesion patterns of other neuropsychiatric symptoms in AD. The degree of damage to different regions varies with different symptoms, while the anterior cingulate cortex (black box) is an area of common damage for all symptoms and is the most common damaged area for agitation, irritability, disinhibition, and eating disturbances. In addition, delusions are closely associated with damage to the orbitofrontal and superior temporal lobes, followed by the occipital and other areas of the frontotemporal lobes. Hallucinations are associated with damage to the left superior frontal lobe, followed by the occipital, parietal, and dorsolateral prefrontal lobes. Agitation is associated with damage to the posterior cingulate gyrus, followed by the middle cingulate gyrus and insula. Irritability is closely associated with damage to the right insula. Disinhibition is also closely associated with damage to the insula and the middle frontal lobe, cingulate other regions. Aberrant motor behaviour and eating disturbances mainly affect the orbitofrontal area, and sleep disturbances are also associated with the right middle frontal gyrus and hypothalamus

We summarized the neuroimaging findings of NPSs in AD (Table 4), of which approximately two-thirds used the NPI scale to evaluate NPSs and three-quarters used the NICDS-ADRDA criteria for the diagnosis of AD. Based on the reports we identified for this review, we defined the frequency of the lesion regions in the NPS-specific brain lesion pattern, and the high frequency represented that the region was most affected by the symptom (Figs. 2, 3, 4 and 5, sFigure 2–5). “Lesion” was defined as a pathological lesion associated with NPSs, including gray matter volume atrophy, cortical thinning, decreased white matter integrity, decreased metabolism, and increased Aβ deposition. In addition, brain circuits for apathy, depression, and anxiety in AD patients were also reviewed in this section.

**Table 4:** Detailed brain changes correlates of neuropsychiatric symptoms in AD

NPS items	Neuroimaging markers	Author, year	Subject (Number)	Diagnostic criteria	Scale	Structures associated	References no.
Apathy	Atrophy	Apostolova, 2007	AD (*N* = 35)	NINCDS-ADRDA	NPI	Bilateral anterior cingulate and left medial frontal cortex	[45]
		Bruen, 2008	Mild AD (*N* = 31)	NINCDS-ADRDA	NPI	Anterior cingulate and frontal cortex bilaterally, the head of the left caudate nucleus and in bilateral putamen	[46]
	Cortical thinning	Donovan, 2014	HC (*N* = 229), MCI (*N* = 395), AD (*N* = 188)	NINCDS-ADRDA	NPI	Inferior temporal region	[47]
		Apostolova, 2007	AD (*N* = 35)	NINCDS-ADRDA	NPI	Left cingulate	[45]
		Tunnard, 2011	AD (*N* = 111)	NINCDS-ADRDA and DSM- IV criteria	NPI	Left caudal anterior cingulate cortex and left lateral orbitofrontal cortex, as well as left superior and ventrolateral frontal regions	[48]
	FA	Tighe, 2012	MCI (*N* = 22), mild AD (*N* = 23)	NINCDS-ADRDA, CDR	NPI	Anterior cingulum	[49]
		Kim, 2011	Very mild or mild probable AD (*N* = 51)	NINCDS-ADRDA	NPI	Left anterior cingulum	[50]
		Ota, 2012	AD (*N* = 21)	NINCDS-ADRDA	Apathy Scale	Right anterior cingulate, right thalamus, and bilateral parietal regions	[51]
	Aβ	Mori, 2014	Aβ-positive AD (*N* = 28)	NINCDS-ADRDA	NPI	Bilateral frontal and right anterior cingulate	[52]
	Hypometabolism	Marshall, 2007	AD (*N* = 41)	NINCDS-ADRDA	NPI	Bilateral anterior cingulate region extending inferiorly to the medial orbitofrontal region and the bilateral medial thalamus	[53]
		Holthoff, 2005	AD (*N* = 53)	NINCDS-ADRDA	NPI	Left orbitofrontal regions	[54]
	Neurofibrillary tangle	Marshall, 2006	AD (*N* = 29)	CERAD	NPI	Anterior cingulate	[55]
		Tekin, 2001	AD (*N* = 31)	CERAD	NPI	Left anterior cingulate	[56]
Depression	Atrophy	Son, 2013	AD (*N* = 49)	DSM IV-TR criteria	GDS	Left inferior temporal gyrus	[40]
		Zahodne, 2013	MCI (*N* = 334)	Petersen criteria	NPI	Anterior cingulate cortex	[57]
		Morra, 2009	AD (*N* = 100), MCI (*N* = 200), HC (*N* = 100)	NINCDS-ADRDA, CDR	GDS	Right hippocampal	[58]
	Cortical thickness	Lebedev, 2014	Mild AD/LBD (*N* = 71)	NINCDS-ADRDA	MADRS	Prefrontal and temporal areas	[59]
		Zahodne, 2013	MCI (*N* = 334)	Petersen criteria	NPI	Entorhinal cortex	[57]
		Lebedeva, 2014	AD (*N* = 189)	NINCDS-ADRDA, DSM-IV/ICD-10	CSDD, GDS	Left parietal and temporal brain regions, including supramarginal, superior and inferior temporal and fusiform gyri, right posterior cingulate and precuneus	[60]
	Gray matter hypodensities	Brommelhoff, 2011	AD (*N* = 192)	NINCDS-ADRDA	History of depression	Caudate nucleus and lentiform nucleus	[61]
	White matter atrophy	Lee, 2012	MCI (*N* = 243)	Petersen	NPI	Frontal, parietal, and temporal	[62]
	Aβ	Chung, 2015	aMCI (*N* = 78)	Petersen	GDS/NPI	bilateral frontal cortex	[63]
	Hypometabolism	Lee, 2017	MCI (*N* = 36)	Petersen	HRSD	Right superior frontal gyrus	[64]
		Hirono, 1998	AD (*N* = 53)	DSM-IV, NINCDS-ADRDA	NPI	Bilateral superior frontal and left anterior cingulate cortices	[65]
		Holthoff, 2005	AD (*N* = 53)	NINCDS-ADRDA	NPI	Dorsolateral prefrontal regions.	[54]
Anxiety	Atrophy	Poulin, 2011	Very mild and mild AD (N1 = 90; N2 = 174)	NINCDS-ADRDA	NPI	Amygdala	[66]
		Mah, 2015	aMCI (*N* = 376)	Petersen	NPI	Entorhinal cortical	[67]
		Tagai, 2014	Mild AD (*N* = 26)	NINCDS-ADRDA	Behave-AD	Right precuneus and inferior parietal lobule	[68]
		Nour, 2020	AD (*N* = 35)	NINCDS-ADRDA	NPI	left parahippocampal, posterior cingulate gyrus, left insula and bilateral putamen	[69]
	White matter hyperintensities	Berlow, 2010	AD (*N* = 37)	NINCDS-ADRDA	NPI	–	[70]
		Bensamoun, 2016	HC (*N* = 230), MCI (*N* = 308), AD (*N* = 119)	NINCDS-ADRDA	NPI	All diagnostic groups: frontal, cingulate, and global cerebral; MCI subgroup: frontal and global cerebral	[71]
	Hyperperfusion	Tagai, 2014	Mild AD (*N* = 26)	NINCDS-ADRDA	Behave-AD	Bilateral anterior cingulate cortices	[68]
		Hashimoto, 2006	AD (*N* = 41)	NINCDS-ADRDA	NPI	Bilateral entorhinal cortex. Anterior parahippocampal gyrus, left anterior superior temporal gyrus and insula	[72]
Delusion	Atrophy	Serra, 2010	AD (*N* = 27), aMCI (*N* = 19), HC (*N* = 23)	NINCDS-ADRDA	NPI	Right hippocampus	[73]
		Geroldi, 2002	Mild AD (*N* = 41)	Standardized clinical, neuropsychological, and instrumental evaluation	NPI	Left frontal and right temporal lobe	[74]
		Geroldi, 2000	AD (*N* = 41)	Standardized clinical, neuropsychological, and instrumental evaluation	NPI	Right medial temporal lobe	[75]
	Cortical thickness	Whitehead, 2012	AD (*N* = 113)	NINCDS-ADRDA, DSM-IV	NPI	Left medial orbitofrontal and left superior temporal region	[76]
	FA	Nakaaki, 2013	AD (*N* = 25)	NINCDS-ADRDA	NPI	Left parieto-occipital region, body of the corpus callosum, superior temporal gyrus	[77]
	WMH	Anor, 2017	AD/VaD (*N* = 180)	NIA-AA	NPI	Right frontal	[78]
	White matter changes	Lee, 2006	AD (*N* = 55)	NINCDS-ADRDA	BRSD	Bilateral frontal, parieto-occipital and left basal gangli	[79]
	Hypermetabolism	Mentis, 1995	AD (*N* = 24), HC (*N* = 17)	NINCDS-ADRDA	Sustained reduplicative delusions of misidentification	Sensory association cortices (superior temporal and inferior parietal)	[80]
		Hirono, 1998	AD (*N* = 65)	NINCDS-ADRDA, DSM-IV	Behave-AD /NPI	Left inferior temporal gyrus	[81]
	Hypometabolism	Mentis, 1995	AD (*N* = 24), HC (*N* = 17)	NINCDS-ADRDA	Sustained reduplicative delusions of misidentification	Paralimbic (orbitofrontal and cingulate areas bilaterally) and left medial temporal areas	[80]
		Sultzer, 2003	AD (*N* = 25)	NINCDS-ADRDA, NIA-AA	Neurobehavioral Rating Scale	Prefrontal and anterior cingulate regions	[82]
		Sultzer, 2014	AD (*N* = 88)	NINCDS-ADRDA, NIA-AA	NPI	Right lateral frontal cortex, orbitofrontal cortex, and bilateral temporal cortex	[34]
		Hirono, 1998	AD (*N* = 65)	NINCDS-ADRDA, DSM-IV	Behave-AD /NPI	Left medial occipital region	[81]
		Mega, 2000	AD (*N* = 20)	NINCDS-ADRDA	NPI	Right and left dorsolateral frontal, left anterior cingulate, and left ventral striatal regions along with the left pulvinar and dorsolateral parietal cortex	[83]
Hallucination	Atrophy	Blanc, 2014	AD (*N* = 78)	NINCDS-ADRDA	NPI	Anterior part of the right insula, left superior frontal gyrus and lingual gyri	[84]
		Holroyd, 2000	AD (*N* = 14)	NINCDS-ADRDA	Subjects/caregiver reported	Occipital lobe	[85]
	Cortical thickness	Donovan, 2014	HC (*N* = 229), MCI (*N* = 395), AD (*N* = 188)	NINCDS-ADRDA	NPI	Supramarginal	[47]
	White matter lesions	Lin, 2006	AD (*N* = 10)	NINCDS-ADRDA	Subjects/caregiver reported	Occipital lobe	[86]
	Hypometabolism	Blanc, 2014	AD (*N* = 78)	NINCDS-ADRDA	NPI	Right ventral and dorsolateral prefrontal area	[84]
	Hypoperfusion	Kotrla, 1995	AD (*N* = 46)	HRSD, DSM-III-R, Behave-AD	HRSD, DSM-III-R, Behave-AD	Parietal lobe	[87]
	Hypoperfusion	Mega, 2000	AD (*N* = 20)	NINCDS-ADRDA	NPI	Right and left dorsolateral frontal, left anterior cingulate, and left ventral striatal regions along with the left pulvinar and dorsolateral parietal cortex	[83]
	Atrophy	Bruen, 2008	Mild AD (*N* = 31)	NINCDS-ADRDA	NPI	Left insula, and in anterior cingulate cortex bilaterally	[46]
		Trzepacz, 2013	AD/MCI (*N* = 462)	NINCDS-ADRDA	NPI	Frontal, insular, amygdala, cingulate, and hippocampal regions	[88]
		Hsu, 2015	AD (*N* = 129), MCI (*N* = 31)	NIA-AA	NPI	Posterior cingulate and parieto-occipital sulcus and sulci of the parietal lobes and precuneus	[89]
	FA	Tighe, 2012	MCI (*N* = 22), mild AD (*N* = 23)	NINCDS-ADRDA, CDR	NPI	Anterior cingulum	[49]
	Increased functional connectivity	Balthazar, 2014	Mild to moderate AD (*N* = 20)	NINCDS-ADRDA	NPI	Anterior cingulate cortex and right insula areas	[90]
	Neurofibrillary tangles	Tekin, 2001	AD (*N* = 31)	CERAD	NPI	Left orbitofrontal cortex and left anterior cingulate	[56]
	Hypometabolism	Weissberger, 2017	AD (*N* = 88)	NINCDS-ADRDA, NIA-AA	NPI	Right temporal, middle, and superior gyri, Right calcarine cortex, Right lingual gyrus, Right fusiform gyrus, Right cuneus, Bilateral cingulate, middle, and posterior	[91]
Irritability	Atrophy	Poulin, 2011	Very mild and mild AD (N1 = 90; N2 = 174)	NINCDS-ADRDA	NPI	Amygdala	[66]
	FA	Tighe, 2012	MCI (*N* = 22), mild AD (*N* = 23)	NINCDS-ADRDA, CDR	NPI	Anterior cingulum	[49]
	Increased functional connectivity	Balthazar, 2014	Mild to moderate AD (*N* = 20)	NINCDS-ADRDA	NPI	Anterior cingulate cortex and right insula areas	[90]
	Aβ	Bensamoun, 2016	HC (*N* = 230), MCI (*N* = 308), AD (*N* = 119)	NINCDS-ADRDA	NPI	All diagnostic groups: frontal, cingulate, parietal and global cerebral; AD:parietal	[71]
	Hypometabolism	Weissberger, 2017	AD (*N* = 88)	NINCDS-ADRDA, NIA-AA	NPI	Right temporal, middle, and superior gyri, Right insula, Right precentral and postcentral gyri, Right frontal, middle, and inferior	[91]
Aberrant Motor Behavior	Atrophy	Poulin, 2011	Very mild and mild AD (N1 = 90; N2 = 174)	NINCDS-ADRDA	NPI	Amygdala	[66]
	Increased functional connectivity	Balthazar, 2014	Mild to moderate AD (*N* = 20)	NINCDS-ADRDA	NPI	Anterior cingulate cortex and right insula areas	[90]
	Hypometabolism	Meguro, 1997	Moderately severe AD (*N* = 10)	NINCDS-ADRDA	Subjects/caregiver reported	Striatum and the frontal and temporal lobes	[92]
	Hypermetabolism	Reilly, 2011	AD (*N* = 135)	DSM-IV	NPI	Orbitofrontal cortex	[93]
	Neurofibrillary tangles	Tekin, 2001	AD (*N* = 31)	CERAD	NPI	Left orbitofrontal cortex	[56]
Euphoria	Increased functional connectivity	Balthazar, 2014	Mild to moderate AD (*N* = 20)	NINCDS-ADRDA	NPI	Anterior cingulate cortex and right insula areas	[90]
Disinhibition	Atrophy	Serra, 2010	AD (*N* = 27)	NINCDS-ADRDA	NPI	Bilateral cingulate and right middle frontal gyri	[73]
	Increased functional connectivity	Balthazar, 2014	Mild to moderate AD (*N* = 20)	NINCDS-ADRDA	NPI	Anterior cingulate cortex and right insula areas	[90]
Sleep disturbance	FA	Tighe, 2012	MCI (*N* = 22), mild AD (*N* = 23)	NINCDS-ADRDA, CDR	NPI	Anterior cingulum	[49]
	Hypometabolism	Liguori, 2017	AD (*N* = 18)	NIA-AA	Polysomnography	Hypothalamic	[94]
	Hyperperfusion	Ismail, 2009	AD (*N* = 55)	NINCDS-ADRDA	NPI, CSDD	Right middle frontal gyrus	[95]
Appetite disturbance	Atrophy	Grundman, 1996	AD (*N* = 58), HC (*N* = 16)	NINCDS-ADRDA	Body mass index	Mesial temporal cortex	[96]
	Hypometabolism	Hu, 2002	AD (*N* = 27)	NINCDS-ADRDA	Body mass index	Anterior cingulate cortex	[97]
	Hypoperfusion	Ismail, 2008	AD (*N* = 66)	NINCDS-ADRDA	NPI	left anterior cingulate and left orbitofrontal cortices	[98]

*Abbreviations*: *AD* Alzheimer’s disease, *aMCI* amnestic mild cognitive impairment, *LBD* Lewy body dementia, *VaD* vascular dementia, *HC* healthy controls, *NPS* neuropsychiatric symptom, *WMH* white matter hyperintensities, *GDS* Geriatric Depression Scale, *MADRS* Montgomery-Asberg Depression Rating Scale, *HRSD* Hamilton Rating Scale for Depression, *Behave-AD* Behavioral Pathology in Alzheimer’s Disease Scale, *CDR* Clinical Dementia Rating, *DSM-IV* Diagnostic and Statistical Manual of Mental Disorders, forth version, *ICD-10* International Statistical Classification of Disease, tenth version, *NIA-AA* National Institute on Aging-Alzheimer’s Association workgroups, *CERAD* Consortium to Establish a Registry for AD

### Apathy in AD

#### Apathy altered anterior cingulate cortex in AD

White matter studies on apathy consistently show that patients with low fractional anisotropy (FA)—a measure representing white matter integrity and information transfer speed—of the anterior cingulate cortex (ACC) are more likely to present with apathy symptoms [49–51]. In addition, FA in the right thalamus and bilateral parietal lobes and white matter hyperintensities in the frontal lobe were correlated with apathy [51]. Gray matter studies on apathy showed that gray matter atrophy in the bilateral ACC and left medial frontal cortex [45]. Moreover, decreased gray matter density in the bilateral ACC, frontal cortex, head of the left caudate nucleus and bilateral putamen [46], and decreased cortical thickness in the left caudal ACC, left orbitofrontal cortex (OFC), left superior, and ventrolateral frontal region [48] and inferior temporal region [47] were all correlated with the severity of apathy.

Positron emission tomography (PET) studies have demonstrated that the AD patients with apathy had glucose hypometabolism in the ACC, OFC, ventral striatum, and medial thalamus [53, 54], higher neurofibrillary tangles burden in the ACC [55, 56], and higher amyloid-β (Aβ) deposition in the bilateral frontal lobe and right ACC [52].

#### Anterior cingulate circuit lesions cause apathy in AD

Apathy has been conceptualized as a motivational barrier or defect in goal-directed behaviour [101]. As reported, the normal motivational behaviour is related to the anterior cingulate-subcortical circuit [102]. The anterior cingulate circuit, linking the ventral striatum to the thalamus via the rostromedial ventrolateral globus pallidus interna and ventral pallidum, originates in the ACC and medial OFC [13, 103, 104]. The disruption of this circuit may be crucially involved in effort-based decision making and executive functions [13]. In particular, lesions to the medial OFC and ventral striatum can lead to the inability to connect emotions with ongoing or upcoming behaviour [105].

#### Summary

Apathy is the most common NPS in AD and has been the focus of past research on NPSs. Although the regions of apathy lesions are not the same in different studies, it is consistently shown that apathy is closely related to changes in the structure and function of the medial frontal cortex and the ACC in AD. Meanwhile, subcortical alterations in the ventral striatum, medial thalamus, and ventral pallidum are also related to apathy. The imaging findings in the regions of apathy lesion supports the association of apathy with anterior cingulate circuit lesions in AD (Fig. 2, sFigure 2).

### Depression in AD

#### Cortical and subcortical limbic brain regions abnormalities

Depression associated gray matter volume atrophy and cortical thinning mainly occur in the frontal and temporal lobes, especially in the left dorsolateral prefrontal, right medial prefrontal, OFC, ACC, and inferior temporal gyrus [40, 57, 59, 60]. The severity of depression was also associated with gray matter changes in the right hippocampal [58], entorhinal [57], left parietal [60], and striatal [61] regions. Similarly, depression causes white matter lesions in the frontal, parietal, and temporal lobes [62]. Hypometabolism in the bilateral superior frontal, left anterior cingulate, and dorsolateral prefrontal regions have been noted in patients with depression [54, 64, 65]. The presence of depression was also associated with the accumulation of Aβ in the frontal lobe in aMCI [63].

#### Frontal-limbic circuits abnormalities and depression in AD

Most research shows that depression is associated with frontal-striatal and subcortical limbic circuits in AD [106, 107]. Mayberg’s frontal-limbic model of depression involves the dorsal, ventral, and rostral compartments [108]. The disturbances in the dorsal compartment, which includes the dorsolateral prefrontal, dorsal ACC, and posterior cingulate cortex, cause attentional and cognitive disturbances. The ventral compartment, which consists of the paralimbic cortical, subcortical, and brainstem regions, is associated with the vegetative and somatic symptoms of depression, such as insomnia and loss of appetite. The rostral ACC connects the dorsal and ventral compartments and plays an important regulatory role in the whole network.

Other researchers believe that the hippocampus is the most common area of structural brain changes in depression [58, 109], and is associated with prefrontal cortex damage. A hippocampal–prefrontal cortex model was proposed, in which the hippocampus—a central part of memory function—regulates mood disorders and cognitive dysfunction in depressed patients [110]. The model also emphasizes the role of the limbic system, such as the cingulate gyrus and amygdala.

#### Summary

Depression may precede a cognitive decline in AD [8] and accelerate the rate of cognitive decline [111]. Both depression and apathy were associated with structural brain changes in the frontal, temporal, and occipital lobes, but apathy was more associated with the anterior cingulate-subcortical circuit, and depression was more associated with neuropathology in the frontal-subcortical limbic circuits in AD (Fig. 3, sFigure 3). The subcortical limbic system of depression mainly includes the hippocampus, amygdala, locus ceruleus, substantia nigra, and hypothalamus [106].

### Anxiety in AD

#### Subcortical brain region lesions in AD anxiety

An anxiety state predicts a decreased entorhinal cortical volume [67] and may be associated with amygdala atrophy [66]. The severity of anxiety was also associated with hyperperfusion of the bilateral ACC, decreased gray matter volume in the right precuneus, inferior parietal, left parahippocampal, posterior cingulate gyrus, left insula, and bilateral putamen lobes [68, 69], and hypometabolism in the bilateral entorhinal, anterior hippocampal, left superior temporal and insula regions [72]. ^18^F-Florbetapir-PET studies show the patients with anxiety had higher Aβ deposition in the precuneus—posterior cingulate, frontal, parietal, anterior cingulate cortex and global cerebral [71]. The locus coeruleus in the hypothalamus is also thought to be the centre of the anxiety-related network [25], and anxiety cells are enriched in the CA1 in the ventral hippocampus [112].

#### Amygdala-medial prefrontal cortex mediated anxiety in AD

Anxiety can be thought of as a set of expected emotional, cognitive, and behavioural changes to the uncertainty of potential future threats, accompanied by fear [113]. The amygdala plays a pivotal role in the transmission and interpretation of fear and anxiety because it receives extensive afferents from the thalamus and extracortical sensory systems and as a subcortical visceral afferent pathway (Fig. 4) [100]. The neuronal interactions between the amygdala and cortical regions, such as the OFC, provide a framework for the initiation of coping behaviors based upon the nature of the threat and prior experiences [114]. Grupe DW and Nitschke JB [115] developed the ‘Uncertainty and Anticipation Model of Anxiety’, which emphasizes that activity in the dorsomedial prefrontal regions and OFC reflects probabilistic estimates of future events and expected costs, respectively, and mainly include the amygdala, bed nucleus of the stria terminalis (BNST), ventromedial prefrontal cortex, OFC, anterior mid-cingulate cortex and anterior insula.

An alternative network is also proposed. Under this network, the hippocampus receives convergent, integrated inputs from all sensory systems through the projections of the entorhinal cortex [116], and it works with the entorhinal cortex on situational fear conditioning. Projections from the hippocampus to the BNST and projections from the BNST to hypothalamic and brainstem sites may be involved in the expression of contextual fear conditioning. Theta oscillations within the hippocampus-amygdala-medial prefrontal cortex circuit are associated with anxious behavior [117]. Both this circuit and the ‘Uncertainty and Anticipation Model of Anxiety’ suggest that the amygdala plays a pivotal role in the assessment of, and response to, danger.

#### Summary

Anxiety is more common in individuals with dementia than in those without dementia [21], and it has been described as a risk factor for AD [118]. Anxiety is primarily associated with damage to the subcortical regions in AD: the amygdala plays an important role in risk assessment and response, the locus coeruleus plays an important role in the efferent response system, and the hypothalamus plays an important role in the integration of autonomic and neuroendocrine responses to threats. The anterior mid-cingulate cortex is closely linked to these brain regions and plays a central role in a series of maladaptive responses to uncertainty (Fig. 4, sFigure 4).

### Delusions and hallucinations in AD

#### Frontotemporal region lesions in AD delusion

Delusions are characterized by asymmetrical brain structure change in the frontal and temporal regions as well as mainly atrophy in the right temporal lobe and left frontal lobe [74, 75, 119]. In addition, delusions are associated with gray matter change in the right hippocampus, left frontal lobe, right frontoparietal cortex, and left claustrum [46, 73], and white matter changes in the bilateral frontal, parieto-occipital region, left basal ganglia, the body of the corpus callosum and the superior temporal gyrus [77–79]. The severity of delusions is associated with hypometabolism in the frontal lobe, especially in the right lateral frontal cortex, ACC and OFC [34, 80, 82], but the association with metabolism in the temporal lobe and occipital lobe is inconsistent. Some studies showed delusions had hypometabolism in the bilateral temporal cortex and the left medial occipital region [34, 81], while other studies showed delusions had higher metabolism in the superior temporal, left inferior temporal gyrus and inferior parietal lobe [80, 81] or no connection [82]. The discrepancies among previous studies are likely due to sex differences. One study showed that women with delusions had frontotemporal atrophy in AD, but men with delusions did not have brain atrophy compared to men without delusions [76].

#### Anterior-posterior neural network lesions in AD hallucination

Hallucinations are associated with atrophy of the gray matter in the anterior right insula, left superior frontal gyrus, lingual gyrus and lateral occipital lobes in AD [47, 84], as well as hypometabolism in the right ventral and dorsolateral prefrontal areas [84]. Patients with hallucinations also had hypoperfusion in the parietal lobe [87]. The damaged brain areas associated with hallucinations mainly include the anterior (e.g., dorsolateral prefrontal area) -posterior (e.g., occipital lobes) neural network and the anterior insula. It is worth noting that these studies do not distinguish between the types of hallucinations. The most common form of hallucinations is visual in AD [120]. Visual hallucinations are mainly caused by atrophy and white matter lesions in the occipital lobe [85, 86]. These lesions are related to the disturbance in the lateral frontal cortex, namely, the ventral visual stream system [121].

#### Summary

Both delusions and hallucinations occur in the middle or late AD stage, and hallucinations may be more common in severe dementia [122]. Compared to delusions, only a limited number of studies have looked specifically at hallucinations in AD, so researchers seem to focus more on delusions (despite conflicting evidence and patients clinging to false beliefs) [123]. The brain lesions of delusions mainly occur in the frontotemporal lobe, accompanied by lateralization and sex differences, while brain lesions of hallucinations mainly occur in the anterior-posterior neural network and anterior insula region (Fig. 5, sFigure 5).

### Other neuropsychiatric symptoms in AD

#### Hyperactivity syndrome in AD

Hyperactivity syndrome includes agitation, disinhibition, irritability, euphoria, and aberrant motor behavior [124], which is related to increased functional connectivity in the anterior cingulate cortex and right insula areas of the salience network [90]. Other neuroimaging findings support this view, especially for the agitation. Diffusion tensor imaging studies showed that irritability and agitation are related to the decreased white matter integrity in the ACC, which is a core component of the salience network [125]. Magnetic resonance imaging studies showed that agitation is related to greater atrophy in the frontal, cingulate, insular, amygdala, and hippocampal regions [46, 88] while aberrant motor behaviour is related to greater atrophy in the amygdala [66]. These are predominantly frontolimbic regions and compose many components of the significance network. PET studies showed that aberrant motor behaviour is associated with hypometabolism of the striatum and frontotemporal lobes and hypermetabolism of the OFC [92, 93]. In addition, a study found that the severity of the agitation is correlated with the atrophy score of the posterior temporal lobe [89].

#### Eating disturbance in AD

AD patients sometimes suffer from eating disturbances and weight loss, and nearly half of patients with AD experience appetite changes in the mild stage [126]. A longitudinal study found that patients had accelerated weight loss as many as 6 years before the diagnosis of AD [127]. A functional neuroimaging study found hypoperfusion of the ACC, OFC, and left middle mesial temporal cortices can predict appetite disturbances [98]. Other neuroimaging studies found weight loss is associated with atrophy of the mesial temporal cortex [96] and hypometabolism of the ACC [97]. In addition, the amygdala and OFC affect the internal balance between hunger and satiety and external motivational control of appetite [128]. Therefore, eating disturbances may be related to the network of the ventral (orbitobasal) frontal cortex, medial temporal cortex, and amygdala in AD.

#### Sleep disturbances in AD

Common sleep disturbances in AD include fragmentation of sleep at night, decreased duration of sleep at night, daytime sleepiness, and inversion of the sleep-wake cycle [129]. The relationship between sleep and AD is complex and bidirectional, and the underlying mechanism is the interaction between sleep and Aβ—sleep disturbances increase the generation of Aβ and decrease the elimination of Aβ; and once Aβ accumulates, there is increased sleep disturbance [130, 131]. Amyloid deposition appears to be associated with decreased sleep quality, but not with changes in sleep quantity in the preclinical stage of AD [132]. Worse sleep quality increases the Aβ burden in the precuneus [133], and a shorter sleep quantity at night increases the Aβ burden in the right hippocampus and thalamus in healthy older peoples [134]. In addition, tau pathology as the second hallmark of AD can also cause sleep disturbances. Sleep regulating areas mainly include the brain stem, thalamus, hypothalamus, midbrain and basal forebrain [135]. Many of these areas show tau pathology at pretangle stages or stages by Braak staging, before any cortical tau or amyloid pathology development [136]. Orexin, as an important sleep-wake regulatory marker, increases in the cerebrospinal fluid in patients with moderate to severe AD and is positively correlated with tau protein levels [137]. Hence, tau pathology may also play an important role in sleep disturbances in AD.

#### Summary

Dysfunction of the orbitofrontal–subcortical circuit is characterized by personality changes including disinhibition, agitation, and irritability; this circuit connects the frontal monitoring systems to the limbic system [104]. Few studies on the relationships between other NPSs and neuroimaging have been conducted in AD, but they all are associated with a lesion in the ACC (Fig. 5, sFigure 5).

### Relationship between NPSs and Alzheimer’s disease

Behavioral and neuropsychiatric symptoms are associated with abnormalities in specific brain regions, such as the prefrontal and subcortical limbic regions, which disrupt the normal balance of neurotransmission. According to neuroimmunoregulation theory, this, in turn, is associated with inflammatory pathways that lead to microglial activation and aggregation and the formation of neurofibrillary tangles, ultimately triggering neuronal loss [138]. In addition, NPSs are related to the dysfunction of various neurotransmitter pathways related to AD, including the dopamine system, the serotonin system, and the cholinergic system [139]. In the current section, we will further discuss the molecular and cellular changes associated with stages of AD progression and their relationship to NPSs.

#### NPSs in preclinical Alzheimer’s disease

NPSs are variable and sporadic throughout the course of the disease, but an important group appears early (Fig. 1), especially emotional symptoms (e.g., depression, anxiety, and apathy), before the clinical diagnosis of cognitive impairment [140, 141].

The presence of microglial activation and inflammatory signals in patients with AD prior to “clinical diagnosis” may explain the occurrence of NPSs in the early stages of the disease. Activation of microglia has been shown to be associated with deficits in social interaction [142]. Meanwhile, apathy, anxiety, depression, and agitation were associated with increased pro-inflammatory cytokines (systemic tumor necrosis factor *α*) detected in the serum of AD patients [143, 144]. Similarly, another study found an association between the levels of diverse cytokines present in CSF of patients with dementia, discovering that anti-inflammatory interleukin-6 (IL-6) cytokine levels were inversely proportional to anxiety scores in AD patients [145]. Importantly, Ledo et al. found that depressive-like behavior induced by Alzheimer’s Aβ oligomers in mice is mediated by inflammation through microglial cell activation in the hippocampus, decreasing 5-HT levels in the hippocampus and prefrontal cortex [146].

Depression is also influenced by the reduction of dopamine and serotonin in the brain, while AD has been associated with loss of serotonergic neurons and a decrease in 5-hydrotryptamine (5-HT) levels in the postmortem brains with this disease [147, 148]. In a healthy brain, dopamine is constantly released into the hippocampus, which links emotional feelings with cognitive processes [149, 150]. In AD, a decrease in dopamine levels coupled with a decrease in serotonin triggers depression, which is regarded as a prodromal symptom of AD. In addition, late-life depression and AD share common genetic factors, including brain-derived neurotrophic factor, apolipoprotein E, interleukin-1, and methylenetetrahydrofolate reductase, while inflammatory pathways are activated in both disorders [151, 152]. In this context, the changes produced by late-life depression seem to have an impact on the hippocampus, inducing inflammatory events that activate microglia, which trigger the overproduction of pro-inflammatory cytokines, as described in earlier time about the conceptual scheme of the neuroimmunomodulation theory [138].

#### NPSs in mild to severe Alzheimer’s disease

As AD progresses, most NPSs present in the early stages of AD become more severe and common, and some psychiatric and behavioral symptoms begin to appear. One hypothesis that has been suggested is that AD progressive cholinergic loss (resulting in a loss of inhibition of the dopamine system), in the context of a relatively spared dopaminergic system, may increase the tendency of AD patients to develop psychosis because of a relative striatal hyperdopaminergia [153]. Available evidence suggested that striatal dopamine (D2/D3) receptors are increased in AD patients with delusional compared with AD patients without delusions [154], and that higher striatal D2 receptors are associated with wandering behavior [92].

In addition, several studies have shown a correlation between serotonin deficiency and NPSs. In patients with AD, lower levels of serotonin1A receptors were associated with more severe depressive symptoms [155], and lower concentrations of serotonin in the temporal cortex were associated with hyperactivity and psychosis [148]. The depressed AD patients showed larger and more extensive reductions in serotonin transporters including the midbrain, nucleus accumbens, and thalamus [156]. Further, the study showed glucose metabolism in the right dorsolateral prefrontal cortex was positively correlated with 5-HT transporter ([11C]-DASB) levels in the striatum in AD patients, suggesting that subcortical serotonergic dysfunction may affect cortical function in regions involved in affective processing such as dorsolateral prefrontal cortex. For example, prefrontal cortex interactions with the hypothalamus mediate reward aspects of eating such as food cravings [157].

In the final stage of AD, the pathology of all NPSs becomes complicated and difficult to treat. Worsening mental symptoms (delusions and hallucinations) cause confusion between reality and morbid fantasies, and patients exhibit severe abnormal motor behaviour, often characterized by scratching, which can lead to recurrent hyperfascial skin infections. These destructive NPSs accelerate the death of AD patients.

#### Summary

We believe that there may be two connective mechanisms between NPSs and AD: (A) NPSs arise as a consequence of AD pathology. AD affects key brain regions of underlying behavior, emotion, or mental, so NPSs may be a direct non-cognitive manifestation of AD neurodegenerative disease [158]. AD-related cognitive decline may also develop into depression, anxiety, or similar NPSs as a psychological response. Other NPSs in the AD stage are caused by AD through reverse causality or psychological responses, and the onset of NPSs will aggravate the pathology of AD. (B) NPSs and AD pathology arise as a consequence of some shared pathologic process. In this case, there is no causal relationship between NPSs and AD pathology, but a third factor, such as brain vascular disease or white matter change, leads to the occurrence of AD and NPSs [159, 160].

## Conclusions

NPSs almost universal existence in the AD, combined with their disabling effects on patients and caregivers, is contrasted by the fact that few effective and safe treatments exist, which is mainly attributed to the following three reasons: (1) Lack of reliable and effective measurement of NPSs in AD; (2) Biomarkers associated with symptom-specific in patients with AD have not yet been developed; (3) The relationship between NPS and the pathological mechanism of AD remains unclear.

The current review provides a good complement to these treatment issues. Firstly, we summarized the detailed scale information, as well as some possible problems in the NPSs measurement process, which may be helpful for accurate assessment of NPSs. Next, we described symptom-general and -specific patterns of brain lesions. The anterior cingulate cortex is a commonly damaged region across all symptoms, and the prefrontal cortex, especially the orbitofrontal cortex, is also a critical region associated with most NPSs. This conclusion was supported by an intervention study, which found that greater reduction in orbitofrontal blood flow has been associated with a greater behavioural response to treatment with donepezil [161].

In contrast, the anterior cingulate-subcortical circuit is specifically related to apathy in AD, the frontal-limbic circuit to depression, and the amygdala circuit to anxiety. Understanding symptom-specific brain lesion networks may help track treatment response for targeted drug therapy. For example, it is important to understand whether observed network changes are the result of functional remodeling of defective networks or reflect the plasticity of compensatory circuitry complement in treatment development. Finally, we elucidated the two possible connective mechanisms between NPSs and AD: etiologic pathways and interactions, and summarized the onset time of NPSs. Different NPSs occur in different disease stages of AD, but most symptoms appear in the preclinical AD or mild cognitive impairment stage and develop progressively, which suggested that the critical treatment window for NPSs should be advanced to the early stage of AD.

There are still some limitations in the study of the pathological mechanism of NPSs in AD patients, and more studies are needed to solve them in the future. Firstly, we found the differences between the subtypes of each symptom in exploring the NPS-specific pathologic mechanisms. For example, in addition to the weight loss caused by the loss of appetite mentioned above, AD patients may also experience increased appetite, difficulty swallowing, and other symptoms of eating disturbances. The relationship between dementia stages and eating disorders may depend on the type of eating disorder. For example, people with mild AD are more likely to experience anorexia, while people with moderate AD have an increased appetite and changes in food preferences and eating habits, and people with severe AD have difficulty swallowing [126]. We suspect that in AD, the relationship between the dementia stage and other NPSs may also differ depending on the subtype of symptoms. Therefore, future studies should further explore the relationship between various symptom subtypes and the severity of dementia in order to better understand the pathological association.

In addition, we should pay attention to the pathological superposition of multiple NPSs. One type of NPSs is unlikely to appear alone and instead is most likely to occur with other types of symptoms in AD [162, 163]. Among patients with dementia, 55% report two or more symptoms, and 44% report three or more symptoms [3]. However, most studies, including some included in the current review, did not adjust the presence of other NPSs when exploring the mechanism of a particular NPS. This is a major limitation of the current review.

Some longitudinal studies showed that individuals with two NPSs had an additional 1.9-fold elevated risk of developing dementia compared with those with zero or one NPS, while those with three or more symptoms had an additional risk of 3 [118] and significantly higher odds of having functional limitations [164]. Another study also showed the number of comorbid NPSs, but not symptom clusters, are associated with an increased risk of dementia [165]. These findings suggest that understanding the comorbid pattern of NPSs will help us to further clarify the pathogenesis of NPSs in AD and contribute to clinical evaluation. However, there are few neuroimaging studies on comorbid NPSs and future studies should focus on this issue.

## Supplementary Information

**Additional file 1.**

## Data Availability

Not applicable.

## Abbreviations

AD: Alzheimer’s disease
NPSs: Neuropsychiatric symptoms
CSDD: Cornell Scale for Depression in Dementia
BEHAVE-AD: The Behavioral Pathology in Alzheimer’s disease rating scale
BRSD: Behavior Rating Scale for Dementia
HAMD: Hamilton depression scale
DSS: Depressive Signs Scale
HAMD: Hamilton Depression Scale
HAMA: Hamilton Anxiety Scale
NPI: Neuropsychiatric Inventory
FA: Fractional anisotropy
ACC: Anterior cingulate cortex
OFC: Orbitofrontal cortex
PET: Positron emission tomography
Aβ: Amyloid-β
BNST: Bed nucleus of the stria terminalis
MCI: Mild cognitive impairment
rACC: Rostral anterior cingulate
BG: Basal ganglia
Th: Thalamus
IL-6: Interleukin-6
