# Supplementary material for: Brain mechanisms underlying neuropsychiatric symptoms in Alzheimer’s disease: a systematic review of symptom-general and –specific lesion patterns
Source: Mol Neurodegener. 2021 Jun 7;16:38. doi: 10.1186/s13024-021-00456-1 (PMC8186099; doi:10.1186/s13024-021-00456-1)
Supplement: Supplementary file 1 — Additional file 1. [file 13024_2021_456_MOESM1_ESM.docx]

**Supplementary mateials**

sTable 1. The onset time of neuropsychiatric symptoms: statistics of research findings.

sTable 2. NPSs and cognition dysfunction: statistics of research findings.

sFigure 1. Flow diagram of the screening process according to PRISMA guidelines

sFigure 2. The brain lesion pattern of apathy in AD.

sFigure 3. The brain lesion pattern of depression in AD.

sFigure 4. The brain lesion pattern of anxiety in AD.

sFigure 5. The brain lesion pattern of other neuropsychiatric symptoms in AD.

**sTable 1. The onset time of neuropsychiatric symptoms: statistics of research findings.**

|  | **aMCI vs Controls** | **Mild AD vs aMCI** | **Moderate AD vs**  **Mild AD** | **Severe AD vs Moderate AD** |
| --- | --- | --- | --- | --- |
| Apathy | 2 | 1 |  |  |
| Depression | 1 | 2 |  |  |
| Anxiety | 2 | 2 |  |  |
| Delusion |  | 2 | 1 | 1 |
| Eating disturbance |  | 1 |  |  |
| Sleep disturbance | 3 | 1 | 1 |  |
| Agitation | 1 | 3 | 1 |  |
| Disinhibition |  |  | 1 |  |
| Hallucination |  |  | 2 | 2 |
| Irritability | 1 | 2 |  |  |
| Euphoria |  |  | 1 | 1 |
| AMB | 1 |  | 1 | 2 |

The Numbers in the table represent the number of studies indicating this result. For example, the first number, "2," represents two studies showing the prevalence of apathy was significantly higher in aMCI than in controls. Abbreviations: AMB, Abnormal motor behavior; AD, Alzheimer’s disease; aMCI, amnestic mild cognitive impairment.

**sTable 2. NPSs and cognition dysfunction: statistics of research findings.**

|  | **Executive** | **Language** | **Memory** | **Visuospatial** | **Reasoning** | **Comportment** | **Conceptualization** |
| --- | --- | --- | --- | --- | --- | --- | --- |
| Delusion | 1 | 2 | 2 | — | 1 | — | 1 |
| Hallucination | — | 2 | 1 | 1 | — | — |  |
| Agitation | 3 | 2 | 2 | 1 | — | — | 1 |
| Depression | 1 | — | 1 | — | — | — | — |
| Anxiety | — | — | — | — | — | — | — |
| Euphoria | — | — | — | — | — | — | — |
| Apathy | 4 | — | — | — | — | 2 | — |
| Disinhibition | 2 | — | — | — | — | — | — |
| Irritation | — | — | — | — | — | — | — |
| AMB | 1 | 1 | — | — | — | — | — |
| Sleep | — | — | 1 | — | — | — | — |
| Eating | — | — | — | — | — | — | — |

The Numbers in the table represent the number of studies indicating poorer cognitive function in AD patients with NPSs. For example, the first number, "1," represents one study showing that delusion is related to executive function. Comportment represents social behavior. The dash mean no significant results in previous study. Abbreviation: AMB, abnormal motor behavior.

**sFigure**

**sFigure 1. Flow diagram of the screening process according to PRISMA guidelines**

**
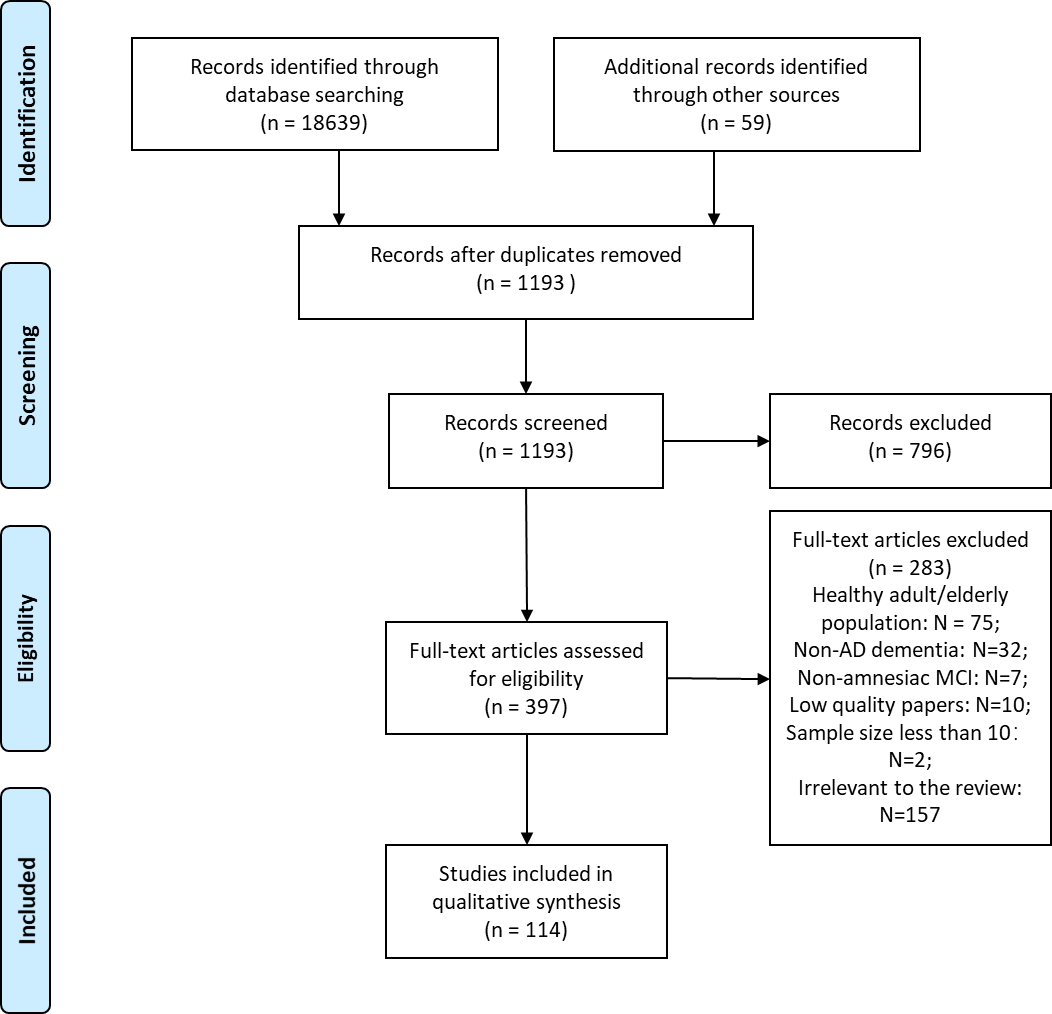
**

**sFigure 2. The brain lesion pattern of apathy in AD.**

**
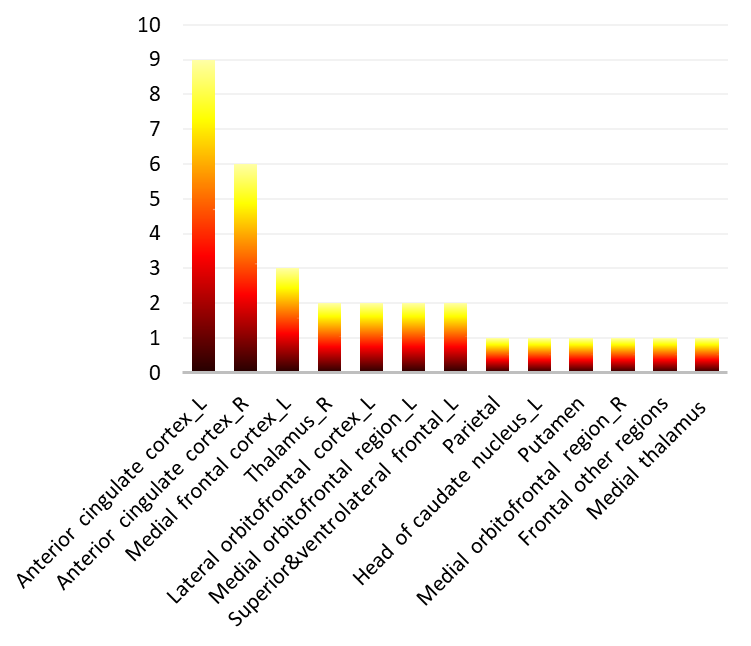
**

Ordinate represent the number of studies indicating apathy is associated with the brain regions, namely the lesion frequency of brain regions. Abbreviations: L, left; R, right.

**sFigure 3. The brain lesion patterns of depression in AD.**

**
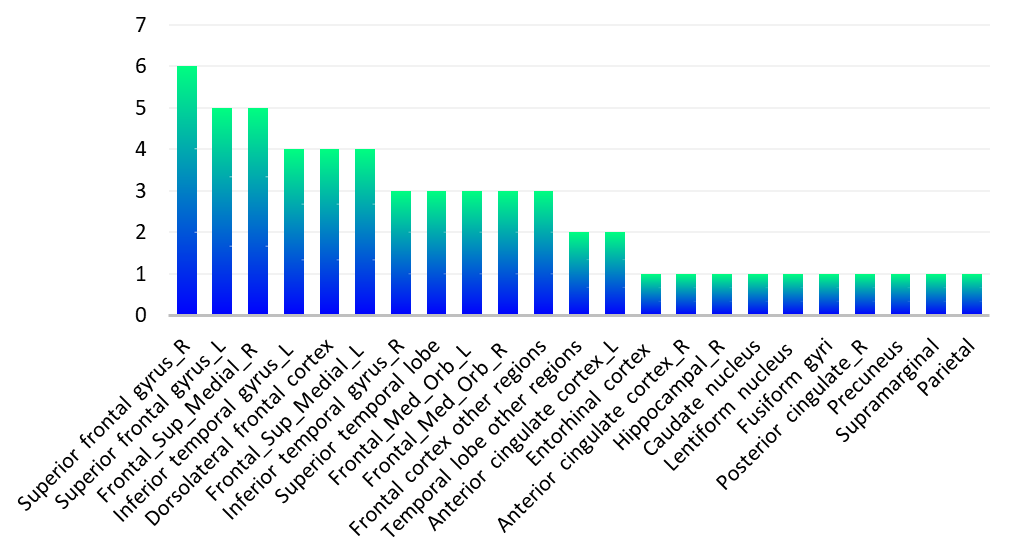
**

Ordinate represent the number of studies indicating depression is associated with the brain regions, namely the lesion frequency of brain regions. Abbreviations: L, left; R, right.

**sFigure 4. The brain lesion patterns of anxiety in AD.**

**
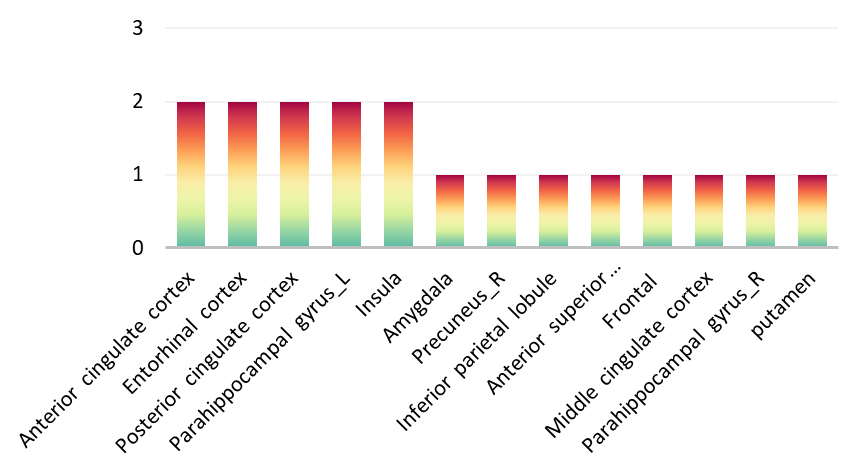
**

Ordinate represent the number of studies indicating anxiety is associated with the brain regions, namely the lesion frequency of brain regions. Abbreviations: L, left; R, right.

**sFigure 5. The brain lesion patterns of other neuropsychiatric symptoms in AD.**

1. **The brain lesion pattern of delusion**

**
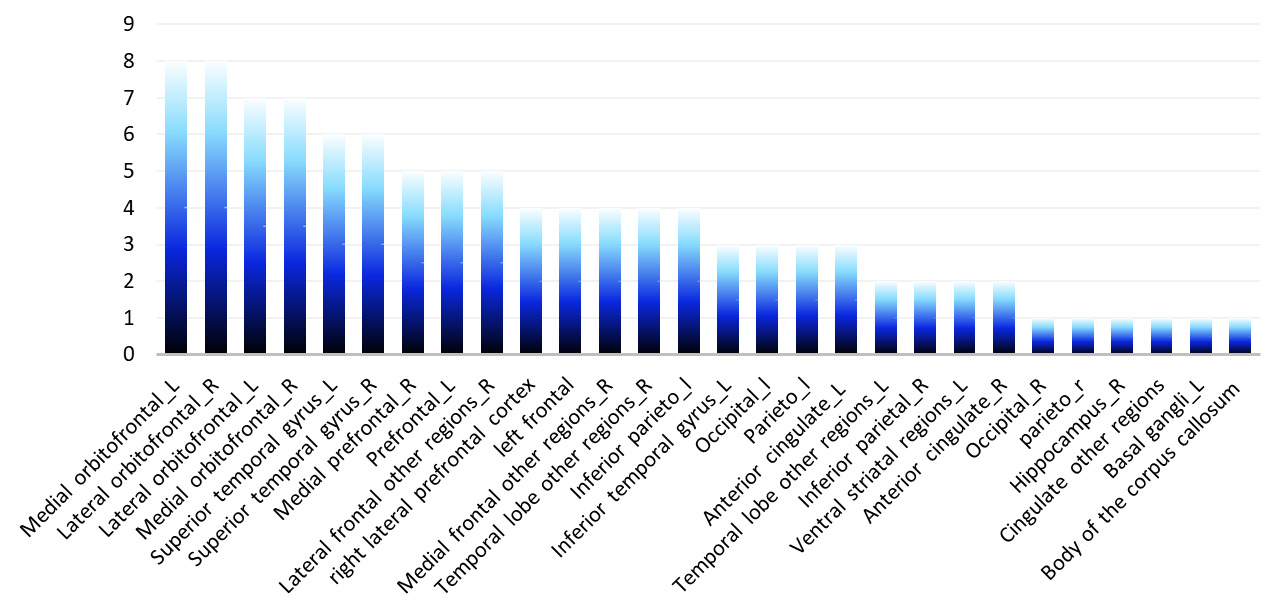
**

1. **The brain lesion pattern of hallucination**

**
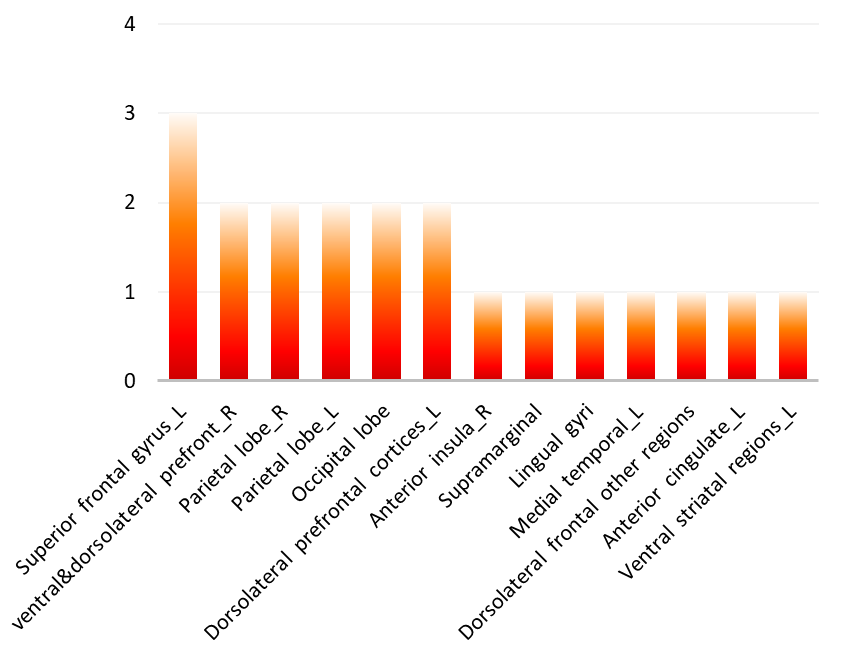
**

1. **The brain lesion pattern of agitation**

**
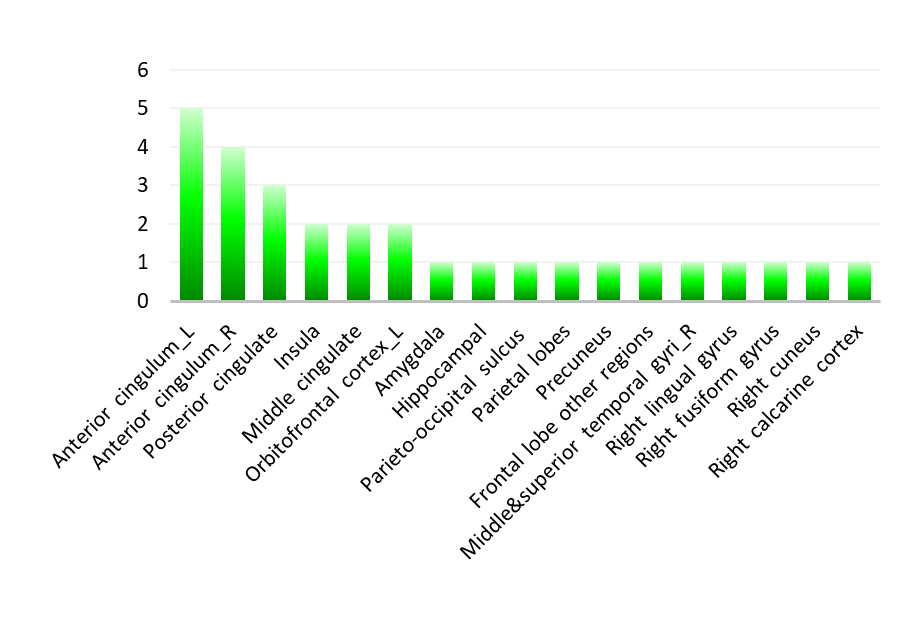
**

1. **The brain lesion pattern of irritability**

**
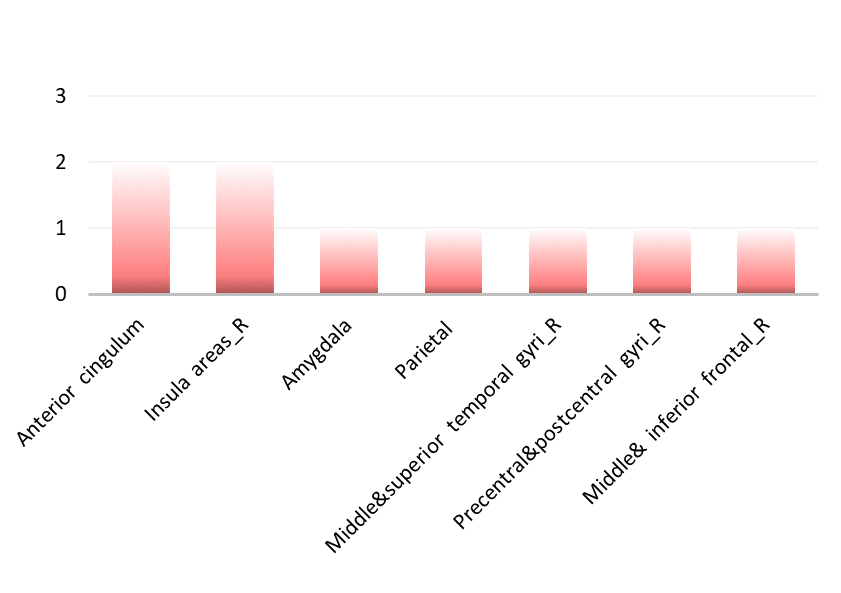
**

1. **The brain lesion pattern of aberrant motor behavior**

**
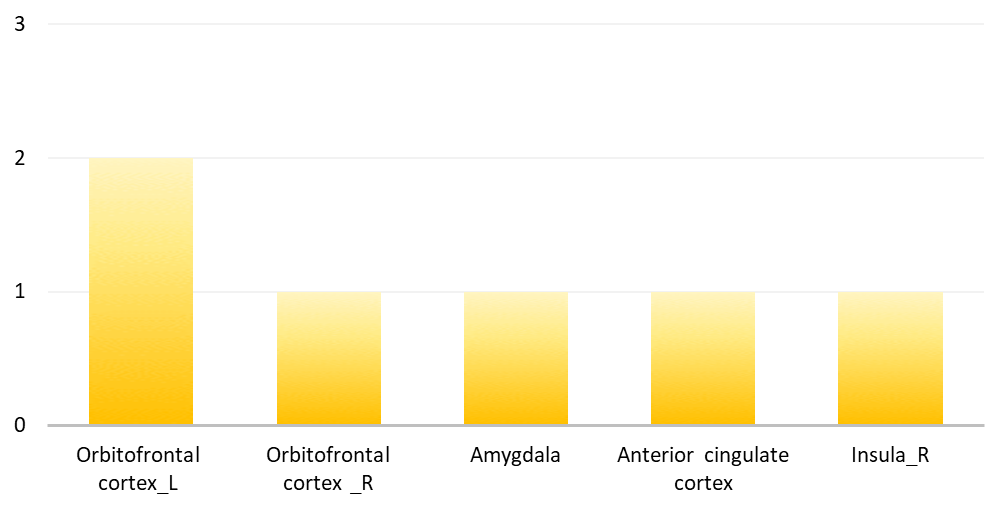
**

1. **The brain lesion pattern of euphoria**

**
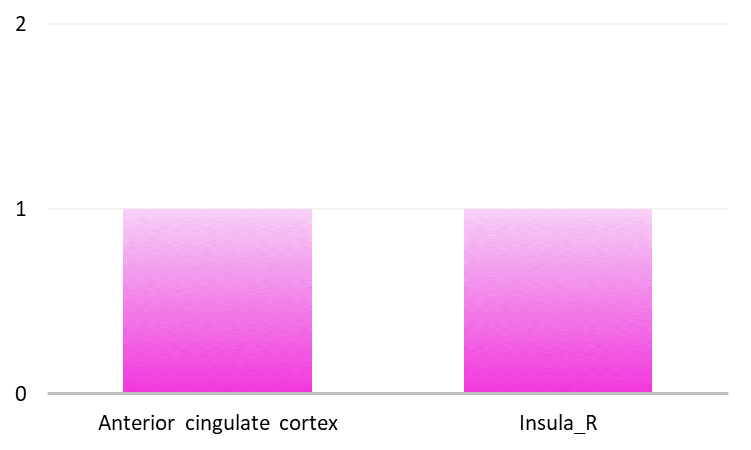
**

**G． The brain lesion pattern of disinhibition**

**
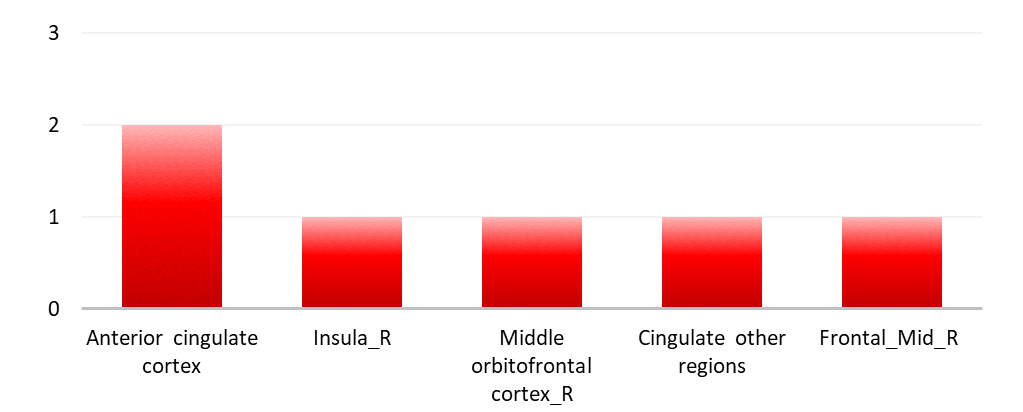
**

**H． The brain lesion pattern of sleep disturbance**

**
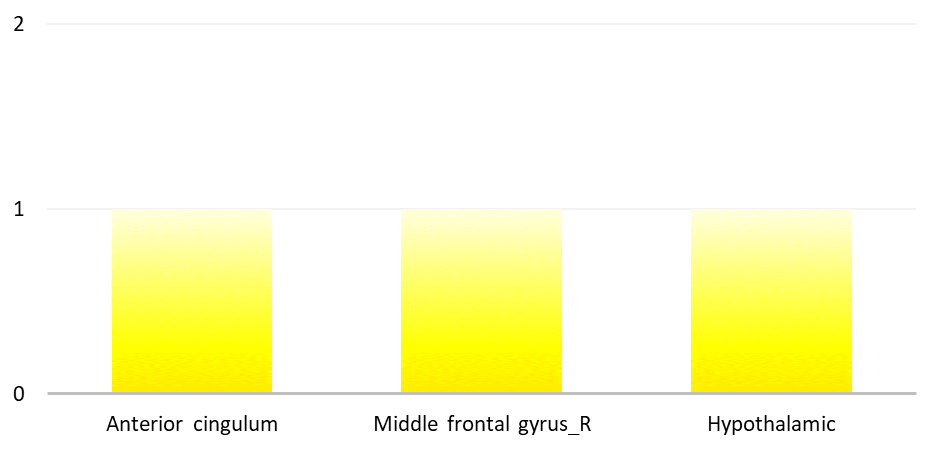
**

**I． The brain lesion pattern of eating disturbance**

**
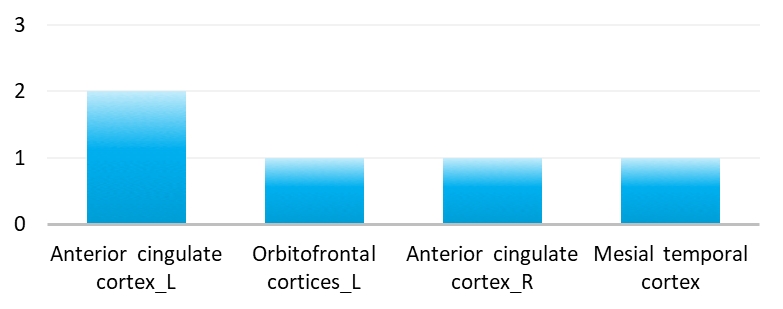
**

Ordinate represent the number of studies indicating the symptom is associated with the brain regions, namely the lesion frequency of brain regions. Abbreviations: Mid, middle; L, left; R, right.
